# *Pseudomonas aeruginosa* Alginate Overproduction Promotes Coexistence with *Staphylococcus aureus* in a Model of Cystic Fibrosis Respiratory Infection

**DOI:** 10.1128/mBio.00186-17

**Published:** 2017-03-21

**Authors:** Dominique H. Limoli, Gregory B. Whitfield, Tomoe Kitao, Melissa L. Ivey, Michael R. Davis, Nora Grahl, Deborah A. Hogan, Laurence G. Rahme, P. Lynne Howell, George A. O’Toole, Joanna B. Goldberg

**Affiliations:** a Department of Microbiology & Immunology, Geisel School of Medicine at Dartmouth, Hanover, New Hampshire, USA; b Department of Pediatrics, Emory University School of Medicine, Atlanta, Georgia, USA; c Department of Biochemistry, University of Toronto, Toronto, Ontario, Canada; d Department of Microbiology and Immunology, Harvard Medical School, Boston, Massachusetts, USA; e Department of Surgery, Massachusetts General Hospital and Harvard Medical School, Boston, Massachusetts, USA; f Shriners Hospitals for Children Boston, Boston, Massachusetts, USA; g Department of Microbiology, Immunology and Cancer Biology, University of Virginia, Charlottesville, Virginia, USA; University of Washington

**Keywords:** *Pseudomonas aeruginosa*, *Staphylococcus aureus*, biofilm, cystic fibrosis, mucoid, polymicrobial

## Abstract

While complex intra- and interspecies microbial community dynamics are apparent during chronic infections and likely alter patient health outcomes, our understanding of these interactions is currently limited. For example, *Pseudomonas aeruginosa* and *Staphylococcus aureus* are often found to coinfect the lungs of patients with cystic fibrosis (CF), yet these organisms compete under laboratory conditions. Recent observations that coinfection correlates with decreased health outcomes necessitate we develop a greater understanding of these interbacterial interactions. In this study, we tested the hypothesis that *P. aeruginosa* and/or *S. aureus* adopts phenotypes that allow coexistence during infection. We compared competitive interactions of *P. aeruginosa* and *S. aureus* isolates from mono- or coinfected CF patients employing *in vitro* coculture models. *P. aeruginosa* isolates from monoinfected patients were more competitive toward *S. aureus* than *P. aeruginosa* isolates from coinfected patients. We also observed that the least competitive *P. aeruginosa* isolates possessed a mucoid phenotype. Mucoidy occurs upon constitutive activation of the sigma factor AlgT/U, which regulates synthesis of the polysaccharide alginate and dozens of other secreted factors, including some previously described to kill *S. aureus*. Here, we show that production of alginate in mucoid strains is sufficient to inhibit anti-*S. aureus* activity independent of activation of the AlgT regulon. Alginate reduces production of siderophores, 2-heptyl-4-hydroxyquinolone-*N*-oxide (HQNO), and rhamnolipids—each required for efficient killing of *S. aureus*. These studies demonstrate alginate overproduction may be an important factor driving *P. aeruginosa* coinfection with *S. aureus*.

## IMPORTANCE

Numerous deep-sequencing studies have revealed the microbial communities present during respiratory infections in cystic fibrosis (CF) patients are diverse, complex, and dynamic. We now face the challenge of determining the influence of these community dynamics on patient health outcomes and identifying candidate targets to modulate these interactions. We make progress toward this goal by determining that the polysaccharide alginate produced by mucoid strains of *P. aeruginosa* is sufficient to inhibit multiple secreted antimicrobial agents produced by this organism. Importantly, these secreted factors are required to outcompete *S. aureus*, when the microbes are grown in coculture; thus we propose a mechanism whereby mucoid *P. aeruginosa* can coexist with *S. aureus*. Finally, the approach used here can serve as a platform to investigate the interactions among other CF pathogens.

## INTRODUCTION

Cystic fibrosis (CF) is the most common fatal, inherited disease among Caucasians. Progressive decline in pulmonary function, resulting from decreased mucociliary clearance, persistent bacterial infections of the airway, and neutrophil-dominated inflammation, is the predominant cause of morbidity and mortality for CF patients (1). CF respiratory infections are notable for their complex polymicrobial nature and recalcitrance to antimicrobial therapeutics. Patients are colonized throughout their lives with a diverse community of pathogens of viral, bacterial, and fungal origins (2). Unfortunately, these infections are rarely eradicated, and patients suffer from frequent exacerbations, hospitalization, and often ineffective treatments with intravenous and inhaled antibiotics. While the importance of interspecies interactions during infection is increasingly appreciated, most studies are still performed with single microbial species in culture, and our knowledge of polymicrobial interactions is limited.

*Pseudomonas aeruginosa* and *Staphylococcus aureus* are two of the most prevalent and often the most problematic pathogens in CF. Both *S. aureus* and *P. aeruginosa* exhibit intrinsic and acquired antibiotic resistance, making these infections difficult to treat (3, 4). *S. aureus* is among one of the earliest pathogens to infect pediatric CF patients, whereas *P. aeruginosa* infections are intermittent early on until a dominant clone emerges and *P. aeruginosa* becomes the predominant pathogen later in life (5). This inverse pattern of infection has led many investigators to speculate that *P. aeruginosa* eliminates *S. aureus* during infection—perhaps outcompeting *S. aureus* for limited nutrients in the lung and/or producing antimicrobial factors to kill *S. aureus* directly (recently reviewed in reference 6). These hypotheses are supported by several *in vitro* studies that demonstrate *P. aeruginosa* can inhibit the growth or reduce the viability of *S. aureus* through multiple mechanisms, including sequestration of iron and inhibition of *S. aureus* respiration via production of the secondary metabolite 2-heptyl-4-hydroxyquinoline-*N*-oxide (HQNO) (7–16).

Importantly, despite evidence that *P. aeruginosa* outcompetes *S. aureus in vitro*, CF patients acquire coinciding pulmonary infections with *P. aeruginosa* and *S. aureus*. We and others have observed a correlation between coinfection and poor patient outcome, whereby infection with both *P. aeruginosa* and *S. aureus* correlates with increased pulmonary exacerbations and lower baseline forced expiratory volumes of the lung in 1 s (FEV_1_) compared to patients who were monoinfected with only *S. aureus* or *P. aeruginosa* (17–19). Examination of 234 CF patients at Emory+Children's Center for Cystic Fibrosis and Airways Disease Research revealed that 73 patients (31%) were coinfected with *P. aeruginosa* and *S. aureus* (17). Moreover, recent data demonstrate higher rates of infection with *S. aureus* in patients later in life than previously appreciated (20). These observations suggest our currently held model that *P. aeruginosa* outcompetes *S. aureus* during infection is oversimplified. We therefore seek to gain a greater fundamental understanding of how coinciding infections with *P. aeruginosa* and *S. aureus* occur in CF patients in an effort to more effectively eliminate these infections.

One hypothesis to explain coinciding *P. aeruginosa*-*S. aureus* infections in CF patients is spatial segregation of these species during infection, such that *P. aeruginosa* antimicrobials are unable to access and thereby kill *S. aureus* during infection. For example, utilizing a wound-like model of *P. aeruginosa-S. aureus* coinfection, DeLeon et al. established species coexistence for up to 7 days (21). The authors attribute *S. aureus* survival to the spatial separation of these species established during biofilm formation in this model. Moreover, physical separation between *P. aeruginosa* and *S. aureus* microcolonies has been observed in human wound biopsy specimens (22). For CF pulmonary infections, recent studies suggest that *P. aeruginosa* and *S. aureus* may occupy the same airspace during infection. Hogan and colleagues identified *P. aeruginosa* and *S. aureus* in the same lobes of the lung by examining protected brush samples (23), and Wakeman et al. have presented histological evidence of bacteria with the morphological characteristics of *Pseudomonas* and *Staphylococcus* infecting the same niche in explanted lungs from a CF patient (24). However, due to current technical constraints studying *in vivo* infections, if and to what extent meaningful physical interactions occur between these pathogens in the context of CF infections is completely unknown.

We therefore sought to design a study to gain insight into whether *P. aeruginosa* and *S. aureus* may interact during infection and, if so, how such coexistence can occur in light of the observation that *P. aeruginosa* can kill *S. aureus in vitro*. We reasoned that *P. aeruginosa* may adopt phenotypes that limit its antagonism toward *S. aureus*. To test this hypothesis, we first asked if *P. aeruginosa* isolates from CF patients were able to outcompete *S. aureus in vitro*, as previously observed, and importantly if strains from coinfected patients were less competitive than *P. aeruginosa* isolates obtained from patients who were not infected with *S. aureus*. Consistent with this idea, we observed that *P. aeruginosa* isolates from coinfected patients were more permissive to growth with *S. aureus* than *P. aeruginosa* isolates from monoinfected patients. Investigation into the nature of coexistence revealed that the presence of a mucoid phenotype limited *P. aeruginosa* antimicrobial action toward *S. aureus*. We found that mucoidy reduces the production of siderophores, HQNO, and rhamnolipids; each is required for robust killing of *S. aureus* (7–16). Moreover, overproduction of the polysaccharide alginate was sufficient to decrease the production of these key compounds. Together these studies suggest that genotypic and phenotypic modifications of *P. aeruginosa* during infection may contribute to coinfection with *S. aureus*.

## RESULTS

### *P. aeruginosa* isolates from coinfected CF patients are less antagonistic toward *S. aureus* than isolates from monoinfected CF patients.

In a previous study, we investigated the clinical outcome of CF patients during coinfection with *P. aeruginosa* and *S. aureus*. We observed that coinfection correlated with poor patient outcome, including a decline in lung function, compared to monoinfected patients (17). To investigate if there are differences in the isolates from these patients that may contribute to establishing coinfection, we obtained 28 *P. aeruginosa* strains and 20 *S. aureus* strains from patients who were either coinfected or monoinfected from the CF Biospecimen Registry (CFBR) at Emory+Children's Center for Cystic Fibrosis and Airways Disease Research. Note that monoinfection refers to the absence of either *P. aeruginosa* or *S. aureus* during the review year, but other pathogens may be present. Positive cultures for *Burkholderia*, *Stenotrophomonas*, *Achromobacter*, *Acinetobacter*, *Chryseobacterium*, *Klebsiella*, *Streptococcus* spp., *Haemophilus influenzae*, and *Escherichia coli* were recorded, but the frequencies of these other microbes were not sufficiently high to establish significant correlations between the presence of individual microbes and clinical outcomes.

**FIG 1:**
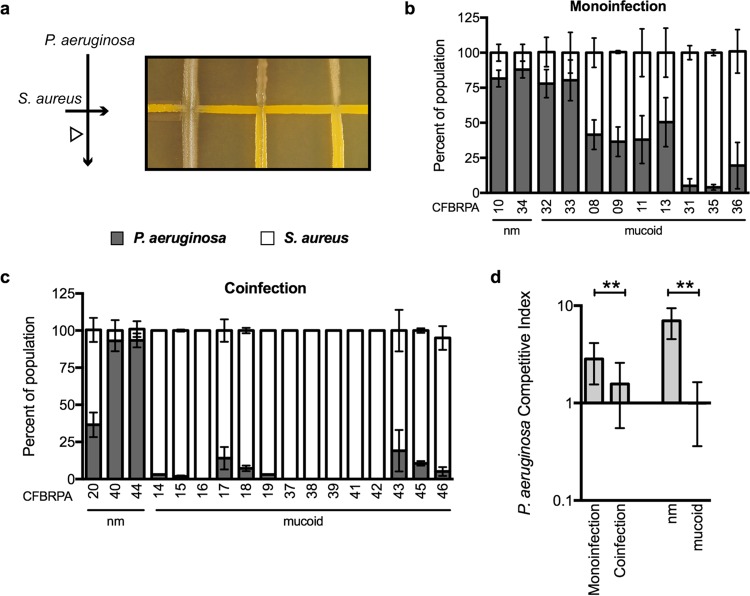
*P. aeruginosa* isolates from coinfected CF patients are less competitive with *S. aureus*. (a) Schematic of the method for cross-streak assay. *S. aureus* (USA300 LAC, JE2) was streaked onto tryptic soy agar followed by cross-streaking with *P. aeruginosa* isolates (CFBRPA) from the CF Biospecimen Registry (CFBR) at Emory+Children's Center for Cystic Fibrosis and Airways Disease Research. Coculture assays were performed by cross-streaking *P. aeruginosa* CF isolates and *S. aureus* on an agar surface, and the percentage of the total population of *P. aeruginosa* (gray bars) and *S. aureus* (white bars) recovered post-cross-streak (white arrowhead in panel a) were enumerated by plating on selective media and dividing the number *of P. aeruginosa* or *S. aureus* CFU by the total CFU (*S. aureus* plus *P. aeruginosa*). *P. aeruginosa* strains from panel b were isolated from CF patients who were infected with only *P. aeruginosa* (monoinfection), and strains in panel c were isolated from patients who were coinfected with *P. aeruginosa* and *S. aureus*. Mucoid and nonmucoid (nm) phenotypes are indicated. In panel d, a summary of the competitive index (CI) of all *P. aeruginosa* strains is indicated according the patient group from which they were isolated or their mucoid phenotype. CI was calculated by dividing the percentage of *P. aeruginosa* by the percentage of *S. aureus* recovered in the post-cross-streak. Error bars indicate standard deviations from three biological replicates performed in triplicate. Statistical significance was determined by performing an unpaired two-tailed *t* test. **, *P* < 0.001.

Based on our previous findings showing worse clinical outcomes for coinfected patients, we then asked the following question: do *P. aeruginosa* and *S. aureus* strains from coinfected patients grow better in coculture than strains from monoinfected patients? To address this question, each *P. aeruginosa* clinical isolate was challenged with a laboratory strain of *S. aureus* (USA300 LAC, JE2) and *S. aureus* clinical isolates with a laboratory strain of *P. aeruginosa* (PAO1) in an agar plate-based cross-streak assay. Single colonies of *S. aureus* and *P. aeruginosa* were cross-streaked according the schematic in Fig. 1a. Note that results were similar regardless of the order in which the pathogens were cross-streaked: i.e., *P. aeruginosa* followed by *S. aureus*, or vice versa. The interactions between the two species was examined by visually inspecting growth at the intersection of the cross-streak and by determining the percentage of the population of *P. aeruginosa* and *S. aureus* by recovering bacterial growth in the poststreak (white arrowhead in Fig. 1a) and enumerating the number of CFU for each *P. aeruginosa* and *S. aureus* strain on selective media (*Pseudomonas* isolation agar [PIA] and mannitol salts agar [MSA], respectively). The *P. aeruginosa* clinical isolates shown in Fig. 1a are representative of the spectrum of *P. aeruginosa* inhibition of *S. aureus*, ranging from most competitive on the left to least competitive on the right.

We found that *P. aeruginosa* clinical strains recovered from patients who were monoinfected with *P. aeruginosa* on average outcompeted *S. aureus* strain JE2 (Fig. 1b), and *P. aeruginosa* strains had a higher competitive index (CI [*P. aeruginosa*/*S. aureus*]) (Fig. 1d, leftmost bar). This pattern of competition was significantly different from that observed for *P. aeruginosa* isolates from patients that were coinfected with *S. aureus* (Fig. 1c), where the CI was on average 2-fold lower (Fig. 1d, second bar). However, when *S. aureus* isolates from CF patients were screened by cross-streaking with *P. aeruginosa* strain PAO1, no difference in the CI for *S. aureus* isolates was observed between mono- and coinfected patients (see Fig. S1 in the supplemental material).

10.1128/mBio.00186-17.1 FIG S1  *S. aureus* cystic fibrosis clinical isolates are not competitive with nonmucoid *P. aeruginosa* PAO1. Cross-streak coculture assays of *S. aureus* isolates (CFBRSA) from the CF Biospecimen Registry (CFBR) with *P. aeruginosa* strain PAO1 were performed by cross-streaking the two species on an agar surface, and the percentages of the populations of *P. aeruginosa* (gray bars) and *S. aureus* (white bars) recovered post-cross-streak were enumerated by plating on selective media and counting the number of CFU, according to the schematic in Fig. 1a. *S. aureus* strains in panel a were isolated from CF patients who were infected with only *S. aureus* (monoinfected), and strains in panel b were isolated from patients who were coinfected with *P. aeruginosa* and *S. aureus*. Error bars indicate the standard deviations of four biological replicates performed in triplicate. Competitive indices were calculated by dividing the percentage of *S. aureus* by the percentage of *P. aeruginosa* recovered post-cross-streak, and a statistically significant difference by an unpaired two-tailed *t* test was not observed between isolates from patients who were monoinfected versus those coinfected. Download FIG S1, TIF file, 0.2 MB. Copyright © 2017 Limoli et al. 2017 Limoli et al. This content is distributed under the terms of the Creative Commons Attribution 4.0 International license.

### Mucoid conversion reduces *P. aeruginosa* inhibition of *S. aureus*.

**FIG 2:**
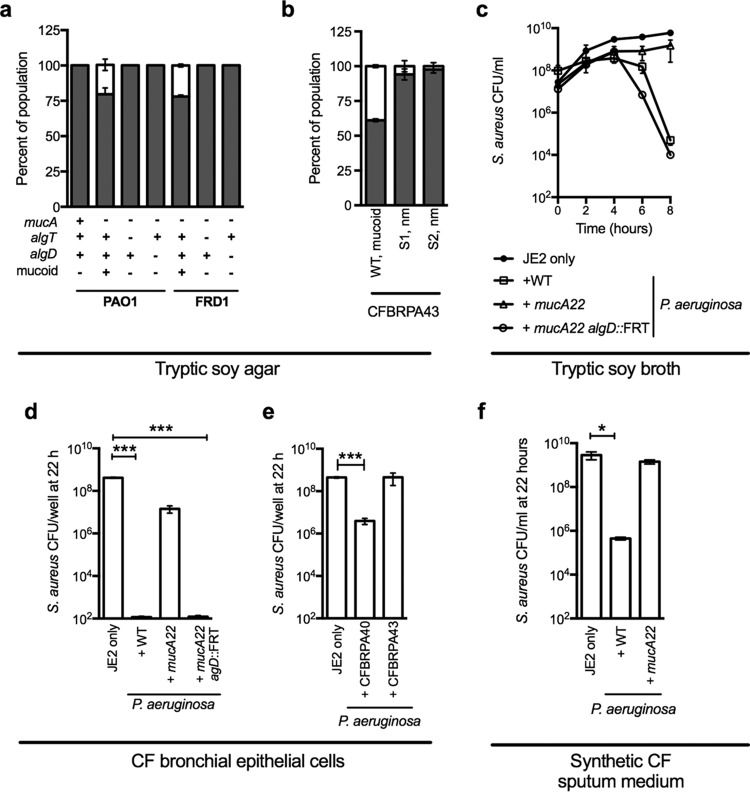
Mucoid conversion prevents *P. aeruginosa* killing of *S. aureus*. *In vitro P. aeruginosa-S. aureus* coculture assays on tryptic soy agar (a and b) and broth (c), in biofilm growth on human CF bronchial epithelial cells (CFBE) (d and e), and in synthetic CF sputum medium (f). In panels a and b, the percentages of the populations of *P. aeruginosa* (gray) and *S. aureus* (white) recovered post-cross-streak are indicated. Isogenic *P. aeruginosa* PAO1 and FRD1 variants, with the *mucA*, *algT*, and *algD* genotypes and the mucoid phenotype indicated below (a), and mucoid *P. aeruginosa* CFBRPA43 and nonmucoid suppressors (S1, nm and S2, nm) of *P. aeruginosa* CFRBPA43 (b) were cross-streaked with *S. aureus* strain JE2. Panel c shows the viable count of *S. aureus* JE2 over time for the indicated strains. In panels d, e, and f, the viability of *S. aureus* JE2 after 22 h of competition with the indicated *P. aeruginosa* strains is indicated. Error bars indicate standard deviations from at least three biological replicates performed in triplicate. Statistical significance was determined by performing an unpaired two-tailed *t* test. ***, *P* < 0.0001.

An additional correlation was revealed during this screen, whereby *P. aeruginosa* isolates that exhibited a mucoid phenotype displayed a CI 6-fold lower on average than that of nonmucoid isolates (Fig. 1d, right 2 bars). Mucoid conversion is generally characterized by overproduction of the polysaccharide alginate, and the designations as nonmucoid and mucoid in Fig. 1 were based on visual observation of alginate production of colonies grown on agar. To test the hypothesis that conversion to the mucoid phenotype promotes *P. aeruginosa* coexistence with *S. aureus*, isogenic *P. aeruginosa* mutants in genes responsible for regulation of alginate synthesis were examined. Mucoid conversion occurs most frequently in *P. aeruginosa* clinical strains via acquisition of mutations in the anti-sigma factor gene *mucA*. In nonmucoid strains, intact MucA sequesters the alternative sigma factor, σ^22^ (AlgT/U), which is the primary regulator of alginate production (25). In mucoid strains, inactivation of MucA (most frequently via the *mucA22* mutation, ΔG430, frameshift) results in release of AlgT, which is now free to activate transcription of the alginate biosynthetic operon initiated at the *algD* promoter (P*algD*) (26). We therefore competed isogenic mutants of the primary regulators of alginate synthesis in *P. aeruginosa* PAO1 with *S. aureus* JE2 to examine the contribution of mucoid conversion to coexistence with *S. aureus* in the cross-streak assay. Consistent with the correlations observed with *P. aeruginosa* clinical isolates, nonmucoid *P. aeruginosa* PAO1 outcompeted *S. aureus*. In contrast, introduction of the *mucA22* mutation in PAO1, which confers a mucoid phenotype, reduced *P. aeruginosa* competition with *S. aureus* (Fig. 2a). Next, to determine if constitutive activation of the sigma factor AlgT as a result of MucA inactivation is responsible for the decreased competition of *P. aeruginosa*, we disrupted the *algT* gene in the *mucA22* mutant and observed restored competition to wild-type (WT) levels. While alginate overproduction is the most visually apparent phenotype in *P. aeruginosa* strains when the *mucA* gene is disrupted, AlgT regulates a number of other factors that have been shown to contribute to competition with *S. aureus*, including genes required for the synthesis of pyoverdine, pyochelin, phenazines, and hydrogen cyanide (27–30). We would therefore hypothesize that AlgT regulates antistaphylococcal factors independent of alginate overproduction. However, when alginate production was selectively removed by disruption of *algD* (the first gene in the alginate biosynthetic operon) in the *mucA22* mutant, *P. aeruginosa* was now able to outcompete *S. aureus*. This result suggests that alginate production may modulate *P. aeruginosa* competition with *S. aureus*. These phenotypes were also reproduced in the mucoid CF isolate FRD1 (*mucA22*) and its nonmucoid derivatives, FRD *mucA22 algT33*::Tn*S1* and FRD *mucA22 algD*::*xylE* (Fig. 2a).

To determine if mutations in *mucA* are responsible for the mucoid phenotype in a representative mucoid isolate from a coinfected patient in this study, the genomic sequence of the *mucA* gene from the clinical *P. aeruginosa* isolate CFBRPA43 was examined. Indeed, strain CFBRPA43 possessed a *mucA22* mutation, which would be predicted to disrupt *mucA* function. To investigate the contribution of mucoid conversion in isolate CFBRPA43, we isolated nonmucoid suppressors by serial passage in broth culture and identified colonies displaying a nonmucoid phenotype. In CF, the most frequent suppressor of mucoid mutations occur within the *algT* gene (31). We therefore sequenced *algT*, confirmed the presence of a mutation in *algT* (S1, 8-nucleotide insertion at position 138; S2, C245A), and compared the competition of two suppressors to the mucoid parental strain in the cross-streak assay. As expected, the nonmucoid variants outcompeted *S. aureus* compared to the mucoid parental strains (Fig. 2b). These data demonstrate that alginate overproduction correlates with the loss of *P. aeruginosa*-mediated inhibition of *S. aureus* and suggest that mucoid conversion may be a factor promoting *P. aeruginosa* and *S. aureus* coexistence during CF respiratory infections.

To determine if *P. aeruginosa* outcompetes *S. aureus* by inhibiting growth or reducing *S. aureus* viability, *P. aeruginosa* and *S. aureus* competition was monitored in shaking broth culture over the course of 8 h by enumerating *P. aeruginosa* and *S. aureus* CFU on selective media (PIA and MSA, respectively) every 2 h. Similar to previous studies, *P. aeruginosa* viability was not altered by the presence of *S. aureus* under any of the conditions examined (10) (see Fig. S2a and b in the supplemental material). For *S. aureus*, coculture with either mucoid or nonmucoid *P. aeruginosa* did not alter viability or growth rate during the initial stages of growth. However, after approximately 4 h of competition, the viability of *S. aureus* drastically decreased in the presence of either nonmucoid strains, PAO1 or PAO1 *mucA22 algD*::FRT (FLP recombination target) (with representative kinetic analysis shown in Fig. 2c). These data indicate that nonmucoid *P. aeruginosa* is capable of killing *S. aureus* during competition and is not simply inhibiting growth. On the other hand, no decrease in viability of *S. aureus* was observed in the presence of the *mucA22* mutant through the duration of the experiment, indicating that alginate-overproducing strains have reduced ability to kill *S. aureus*. Identical results were observed when mucoid and nonmucoid *P. aeruginosa* strains were grown in competition with *S. aureus* methicillin-sensitive strain Newman (Fig. S2c).

10.1128/mBio.00186-17.2 FIG S2  Nonmucoid *P. aeruginosa* outcompetes *S. aureus*. *P. aeruginosa-S. aureus* coculture assays during planktonic growth (a, b, and c) and during biofilm growth on CF bronchial epithelial cells (CFBE [d**]**). In panels a and b, the viability of *P. aeruginosa* strain PAO1 (WT) and isogenic mutants was measured as log_10_ CFU/well over the course of 8 h in monoculture (a) and in coculture with *S. aureus* strain JE2 (b). In panels c and d, the viability of *S. aureus* JE2 was measured as log_10_ CFU/ml after 8 h of coinfection in panel c and CFU/well over 22 h in panel d. Data in panels a, b, and d are representative of three biological replicates, and in panel c, error bars indicate standard deviations from three biological replicates performed in triplicate. Statistical significance was determined by performing an unpaired two-tailed *t* test. ***, *P* < 0.0001. Download FIG S2, TIF file, 0.2 MB. Copyright © 2017 Limoli et al. 2017 Limoli et al. This content is distributed under the terms of the Creative Commons Attribution 4.0 International license.

### Mucoid conversion prevents *P. aeruginosa* killing of *S. aureus* during competition in models of CF respiratory infection.

During CF pulmonary infections, both *P. aeruginosa* and *S. aureus* exist in complex and dynamic communities that influence their intra- and interspecies interactions (32, 33). Recently, we have established an *in vitro* human airway cell—dual bacterial biofilm infection model, whereby *P. aeruginosa* and *S. aureus* form mixed-species biofilms on monolayers of human bronchial epithelial cells homozygous for the ΔF508 cystic fibrosis transmembrane conductance regulator mutation (CFBE) (10, 34). To determine how alginate overproduction influences the dynamics of interspecies interactions in this model, wild-type (WT) PAO1, *mucA22*, and *mucA22 algD*::FRT strains were cocultured with *S. aureus* JE2 on CFBE cells for 22 h, and CFU were enumerated at 6, 18, and 22 h. Similar to the observation in Fig. 2c for broth culture, nonmucoid *P. aeruginosa* (both WT and *mucA22 algD*::FRT strains) coexists with *S. aureus* early during culture, followed by a sharp decrease in *S. aureus* viability by 18 h (representative kinetic CFU illustrated in Fig. S2d in the supplemental material and at the 22-h time point in Fig. 2d). This finding is consistent with a previous report from our group using nonmucoid *P. aeruginosa* strains PAO1 and PA14 cocultured with *S. aureus* strain 8325-4 (10). In contrast, *S. aureus* viability is unaltered for the duration of the experiment when cocultured with the *mucA22* mutant. Additionally, representative nonmucoid (CFBRPA40) and mucoid (CFBRPA43) *P. aeruginosa* clinical isolates, identified to be competitive and noncompetitive with *S. aureus* in the cross-streak assay, respectively (Fig. 1c), were selected for analysis in this model. As predicted, nonmucoid *P. aeruginosa* strain CFRBPA40 reduced the viability of *S. aureus*, compared to mucoid *P. aeruginosa* strain CFBRPA43 (Fig. 2e). Finally, to investigate if nutrient availability in the CF respiratory environment influences competitive dynamics between *P. aeruginosa* and *S. aureus*, coculture growth was monitored in synthetic CF sputum medium (35). In accordance with prior observations, *S. aureus* viability was reduced during growth with nonmucoid *P. aeruginosa* (PAO1), but remained unaltered in the presence of mucoid *P. aeruginosa* (PAO1 *mucA22*) (Fig. 2f). These data demonstrate that mucoid conversion supports *P. aeruginosa*-*S. aureus* coexistence in mixed-species communities during growth in models that mimic the CF respiratory environment.

### Induction of alginate reduces *P. aeruginosa* antimicrobial activity independent of AlgT activation.

**FIG 3:**
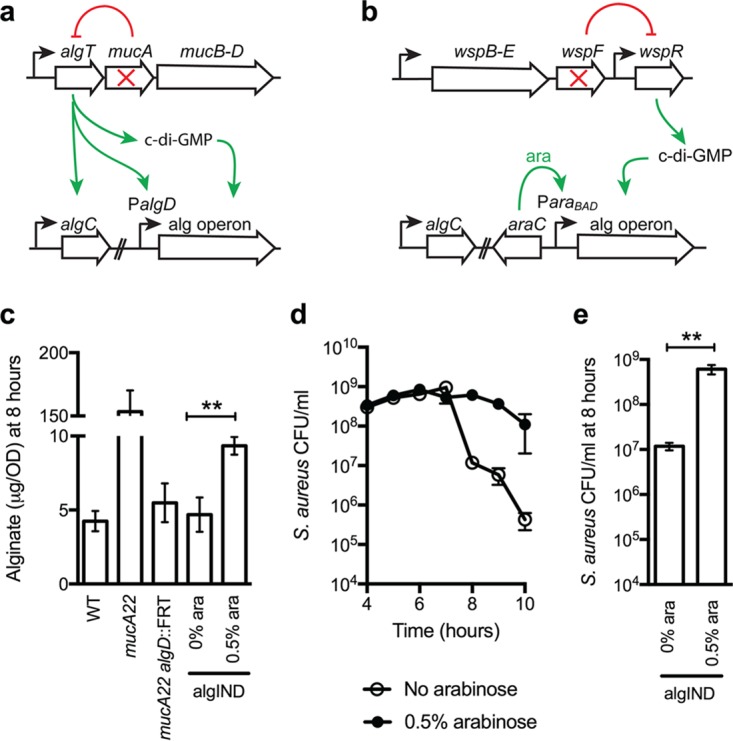
*P. aeruginosa* alginate production promotes coexistence with *S. aureus*. (a) Abbreviated schematic of alginate regulation in *P. aeruginosa*. Disruption of MucA results in release of AlgT, activating transcription of the *algC* gene, genes encoded in the alginate biosynthetic operon (*alg* operon, under control of the *algD* promoter P*algD*), and *bis*-(3′,5′)-cyclic dimeric GMP (c-di-GMP)—all required for alginate synthesis. (b) Schematic of alginate regulation in *P. aeruginosa* PAO1 Δ*wspF* P*algD*::*araC*-P*araBAD* (PAO1algIND), whereby P*algD* was replaced by *araC*-P*araBAD*, to place the *alg* operon under inducible control of arabinose. Deletion of *wspF* results in inhibition of WspR and production of c-di-GMP. In panel c, alginate was extracted from nonmucoid wild-type PAO1 (WT) and the PAO1 *mucA22*, PAO1 *mucA22 algD*::FRT, and PAO1algIND mutants grown without arabinose (0% ara) and with 0.5% arabinose (0.5% ara) and quantified by a standard carbazole assay. In panels d and e, the log_10_ CFU/ml for *S. aureus* JE2 are indicated when grown in the presence of PAO1algIND without (open circles) and with (solid circles) 0.5% arabinose for 10 h (d); *S. aureus* log_10_ CFU/ml at 8 h are shown only in panel e. Error bars indicate standard deviations from four biological replicates performed in triplicate. Statistical significance was determined by performing an unpaired two-tailed *t* test. **, *P* < 0.01.

The data presented thus far demonstrate that mucoid conversion limits the ability of *P. aeruginosa* to reduce *S. aureus* viability; however, whether overproduction of alginate by *P. aeruginosa* is sufficient for this interaction is unclear. Therefore, to study this behavior in more detail, we sought to engineer a *P. aeruginosa* strain whereby alginate production can be modulated in a nonmucoid strain with intact *mucA*. Such a strategy would allow us to assess the influence of alginate overproduction on *P. aeruginosa* physiology and gene expression, without the activation of the entire AlgT regulon. Alginate is a high-molecular-weight acidic polysaccharide composed of nonrepeating subunits of selectively O-acetylated d-mannuronic acid and its C5′ epimer, l-guluronic acid (36). Genes required for alginate biosynthesis are primarily organized within a single operon (*algD*, -*8*, -*44*, -*K*, -*E*, -*G*, -*X*, -*L*, -*I*, -*J*, -*F*, and -*A*), with the exception of the *algC* gene (see the schematic in Fig. 3a). *alg*C encodes a phosphomannomutase required to convert mannose-1-phosphate to mannose-6-phosphate (37). To engineer a strain that bypasses the need for AlgT activation of these genes (schematic in Fig. 3b), we replaced the promoter of the alginate biosynthetic operon (P*algD*) with an arabinose-inducible promoter (*araC*-P*ara*_BAD_) on the chromosome of nonmucoid strain PAO1. Additional regulation of alginate synthesis occurs at the posttranscriptional level, whereby polymerization by the inner membrane proteins Alg8 and Alg44 requires binding of the second messenger *bis*-(3′,5′)-cyclic dimeric GMP (c-di-GMP) to Alg44 (38). In *mucA* mutants, the requisite c-di-GMP would be provided through the action of diguanylate cyclases activated by AlgT. To circumvent the need for AlgT activation, P*algD*::araC-P*ara*_BAD_ was constructed in a mutant with constitutively high levels of c-di-GMP (PAO1 Δ*wspF*) (39).

To determine if PAO1 Δ*wspF* P*algD*::*araC*-P*ara*_BAD_ (abbreviated “PAO1algIND,” for “PAO1 alginate inducible,” from this point forward) produces alginate only in the presence of arabinose under the conditions utilized in the coculture assays, PAO1algIND was grown with and without 0.5% arabinose for 8 h in a shaking broth culture, and the amount of alginate produced was determined by a standard carbazole assay. (Growth rates for both *P. aeruginosa* and *S. aureus* were identical with and without arabinose.) Alginate production increased by approximately 2-fold in the presence of 0.5% arabinose (9.3 µg/optical density at 600 nm [OD_600_]) compared to without arabinose, demonstrating alginate synthesis can be induced using this strategy (Fig. 3c). The increase in alginate production was modest however compared to PAO1 *mucA22* (153.4 µg/OD_600_) (Fig. 3c; note *y* axis). To determine if this level of alginate production is sufficient to prevent *P. aeruginosa*-mediated reduction of *S. aureus* viability, PAO1algIND was grown with and without arabinose in the presence of *S. aureus*. Without arabinose, PAO1algIND reduced the viability of *S. aureus* by ~1,000-fold by 8 h, whereas in the presence of 0.5% arabinose, *S. aureus* viability was maintained for the 10 h of the assay period (Fig. 3d and e). The time required for uninduced PAO1algIND to initiate killing of *S. aureus* was longer than observed for PAO1 (8 h compared to 4 h [Fig. 2c]). Overall, however, the sustained viability of *S. aureus* observed when competed with PAO1algIND plus arabinose supports the previous observation that alginate prevents *P. aeruginosa* killing of *S. aureus*. Furthermore, our analysis of the PAO1algIND strain suggests that the amount of alginate produced by PAO1 *mucA22* is in excess of the amount required to reduce killing of *S. aureus*.

### Alginate overproduction reduces the expression of a subset of *P. aeruginosa* virulence genes.

*P. aeruginosa* secretes several antistaphylococcal effectors which can inhibit respiration (hydrogen cyanide, quinolones, and phenazines) and sequester iron (siderophores) (7–16). To determine how alginate overproduction prevents *P. aeruginosa* from killing *S. aureus*, we asked if alginate overproduction alters the expression profile of genes associated with physiological pathways important for *S. aureus* interactions and infection in the CF airway. We utilized NanoString digital multiplexed gene expression technology (40, 41) to quantify the expression level of *P. aeruginosa* mRNA transcripts in mucoid PAO1 *mucA22* compared to nonmucoid PAO1 *mucA22 algD*::FRT, as well as PAO1algIND grown with and without arabinose. We utilized gene-specific probes described previously (NanoString codeset PAV2) that monitor the expression of 75 transcripts associated with biofilm formation, polysaccharide production, iron acquisition, quorum sensing, and virulence (41). In brief, transcripts were monitored using a set of two hybridization probes complementary to each transcript of interest, with one probe enabling the capture of the transcript and the other containing a unique fluorescent barcode for direct transcript enumeration that reflects abundance in the sample.

**FIG 4:**
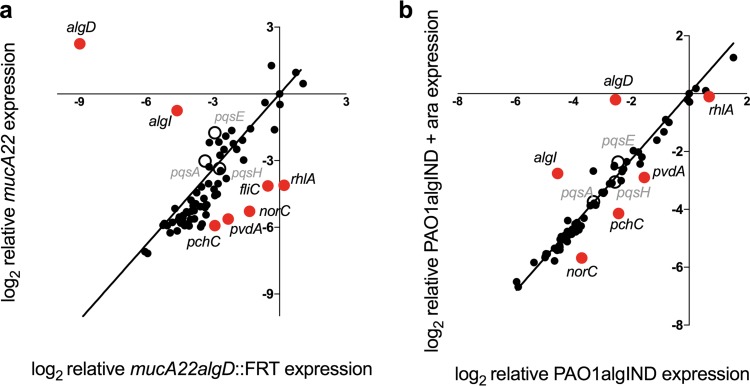
Alginate overproduction decreases the expression of *P. aeruginosa* genes required to reduce *S. aureus* viability. Relative expression of a subset of *P. aeruginosa* virulence genes was compared in PAO1 *mucA22* (mucoid) and PAO1 *mucA22 algD*::FRT (nonmucoid) strains (a) and PAO1algIND strains grown with (mucoid) and without 0.5% arabinose (ara) (nonmucoid) (b) by the NanoString nCounter analysis system. The abundance of 75 transcripts was examined with a custom-designed codeset. Transcripts were log_2_ transformed and normalized to two *P. aeruginosa* housekeeping genes (*rpoD* and *ppiD*). Genes determined to be significantly differentially regulated when alginate is produced by an unpaired *t* test followed by the two-stage linear step-up procedure of Benjamini, Krieger, and Yekutieli (with *q* = 1% for false discovery) are indicated in red. Genes involved in the PQS pathway are indicated with open circles.

To assess changes in *P. aeruginosa* transcript profiles during alginate production, the relative levels of expression for each gene in the PAV2 NanoString codeset were compared between PAO1 *mucA22* and PAO1 *mucA22 algD*::FRT (Fig. 4a), as well as PAO1algIND with and without arabinose to induce alginate production (Fig. 4b). Differences in raw transcript levels were also analyzed by their ranked abundance (heat map shown in Fig. S3a and raw data in Table S1 in the supplemental material). The relative transcript levels of five genes in the codeset were significantly lower (*rhlA*, *norC*, *fliC*, *pvdA*, and *pchC*) in the *mucA22* mutant compared to the *mucA22 algD*::FRT mutant (Fig. 4a). As expected, we observed significantly higher levels of *algD* transcript in the *mucA22* compared to the *mucA22 algD*::FRT strain. However, a larger amount of *algI* was also observed. This observation could be a result of inactivation of downstream genes in the alginate biosynthetic operon in the *mucA22 algD*::FRT strain or from the acquisition of a secondary mutation in *algT* during the construction of the *algD*::FRT strain. To confirm that *algT* has not been altered in this strain, the sequences of *algT* in PAO1 *mucA22* and PAO1 *mucA22 algD*::FRT were compared and confirmed to be identical. The expression of *algT* and the subset of AlgT-regulated genes examined here were also not significantly different between these strains, confirming that alginate production is reduced in PAO1 *mucA22 algD*::FRT, but it retains constitutive expression of *algT* (see Table S2 in the supplemental material). In concordance with the comparison of the mucoid *mucA22* mutant and its nonmucoid derivative PAO1 *mucA22 algD*::FRT, transcript levels of *rhlA*, *norC*, *pvdA*, and *pchC* were lower in PAO1algIND when grown in the presence of arabinose (and thus producing alginate), compared to without this inducer (Fig. 4b). While transcript levels of *fliC* were also lower, the difference was not statistically significant. The relative expression of *algD* in PAO1algIND in the presence of arabinose was less than the expression in PAO1 *mucA22*, which corresponds to the modest increase in alginate production observed in Fig. 3c for the alginate-inducible strain. Accordingly, a smaller change in the relative expression of the *rhlA*, *pvdA*, *pchC*, and *norC* genes was also observed. Finally, gene expression profiles for *P. aeruginosa* when grown in competition with *S. aureus* were examined, and no significant difference in gene expression was observed (Table S1), as we reported previously using transcriptome sequencing (RNA-Seq) analysis (10).

10.1128/mBio.00186-17.3 FIG S3  Expression analysis of *P. aeruginosa* virulence genes during alginate overproduction. mRNA transcript abundance of a subset of *P. aeruginosa* virulence genes was compared in PAO1 *mucA22* (mucoid) and PAO1 *mucA22 algD*::FRT (nonmucoid) strains by the NanoString nCounter analysis system. (a) A heat map was generated based on the ranked abundance (from 1 to 75) of the raw transcript values for each of the three biological replicates for each strain. Each cluster of genes is labeled with their known or predicted functions. The color gradient (white to purple) indicates the relative expression level of each gene with purple indicating the most highly expressed genes. (b to e) The expression of *pvdA* (b), *pqsL* (c and d), and *rhlA* (e) relative to the housekeeping gene *rpoD* was determined by qRT-PCR for the indicated *P. aeruginosa* strains. The means of biological triplicates (b, c, and e) and quadruplicates (d) and standard deviations are indicated. Statistical significance was determined by one-way ANOVA followed by a Dunnett’s multiple comparison test. *, *P* < 0.05; **, *P* < 0.01. Download FIG S3, TIF file, 78 MB. Copyright © 2017 Limoli et al. 2017 Limoli et al. This content is distributed under the terms of the Creative Commons Attribution 4.0 International license.

10.1128/mBio.00186-17.7 TABLE S1  Raw NanoString data. Download TABLE S1, XLSX file, 0.1 MB. Copyright © 2017 Limoli et al. 2017 Limoli et al. This content is distributed under the terms of the Creative Commons Attribution 4.0 International license.

10.1128/mBio.00186-17.8 TABLE S2  Relative expression of AlgT-regulated genes in PAV2 between PAO1 *mucA22* and PAO1 *mucA22 algD*::FRT. Download TABLE S2, DOCX file, 0.1 MB. Copyright © 2017 Limoli et al. 2017 Limoli et al. This content is distributed under the terms of the Creative Commons Attribution 4.0 International license.

### Alginate overproduction reduces pyoverdine production.

mRNA analysis revealed that expression of genes required for siderophore production (*pvdA* and *pchC*, for the synthesis of pyoverdine and pyochelin, respectively) were lower when alginate was overproduced. These siderophores specifically chelate iron (Fe^3+^), a role previously demonstrated to be involved in *P. aeruginosa* inhibition of *S. aureus* growth (8, 10). We therefore determined if pyoverdine (the predominant *P. aeruginosa* siderophore) plays a role in inhibiting growth of *S. aureus* under the conditions utilized in this study. We first confirmed reduced expression of *pvdA* in the *mucA22* mutant compared to the WT and *mucA22 algD*::FRT strains by quantitative real-time PCR (qRT-PCR) (Fig. S3b).

**FIG 5:**
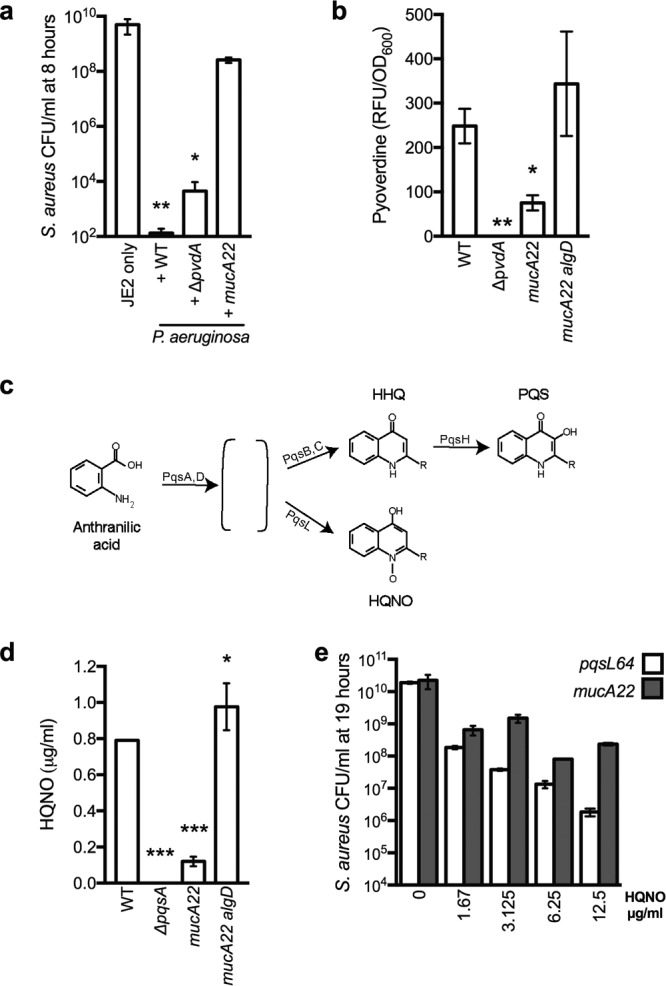
Alginate overproduction inhibits antistaphylococcal exoproducts. (a) *In vitro P. aeruginosa-S. aureus* coculture assays in planktonic culture with the indicated strains. Log_10_ CFU/ml for *S. aureus* JE2 are indicated after 8 h of incubation. (b) Pyoverdine was quantified as relative fluorescence units (RFU)/OD_600_ produced by planktonic *P. aeruginosa* strains grown for 8 h. (c) Schematic of 2-heptyl-4-hydroxyquinoline-*N*-oxide (HQNO) synthesis. The PqsA to -E, -L, and -H enzymes catalyze the synthesis of a series of 4-hydroxy-2-alkylquinolones (HAQs). The conversion of anthranilic acid to uncharacterized intermediates (indicated by the brackets) is catalyzed by PqsA and -D, followed by conversion to either 4-hydroxy-2-heptylquinoline (HHQ) by PqsB and -C (which can be converted to 3,4-dihydroxy-2-heptylquinoline [PQS] by PqsH) or converted to HQNO by PqsL. (d) HQNO quantification by LC-MS from supernatants derived from the indicated strains following 8 h of incubation in planktonic culture and (e) *in vitro P. aeruginosa-S. aureus* coculture assays in planktonic culture with *P. aeruginosa* PAO1 *pqsL64* (white) and PAO1 *mucA22* (gray) in the presence of the indicated concentrations of HQNO. Log_10_ CFU/ml for *S. aureus* JE2 are indicated after 19 h of incubation. Error bars indicate standard deviations from three biological replicates performed in triplicate. In panels a and b, statistical significance was determined by performing an analysis of variance (ANOVA) followed by a Dunnett’s multiple comparison test comparing each condition to the WT in panels a and d (*, *P* < 0.05; **, *P* < 0.01; ***, *P* < 0.0001) and to JE2 only in panel b. In panel e, statistical significance was determined by performing independent ANOVA analyses for PAO1 *pqsL64* and PAO1 *mucA22* followed by a Dunnett’s multiple comparison test comparing the viability of *S. aureus* JE2 in the presence of each HQNO concentration to the condition without HQNO, and the viability was significantly decreased at each concentration: for PAO1 *pqsL64*; *P* ≤ 0.0001, and for PAO1 *mucA22*, *P* ≤ 0.01.

We then competed *P. aeruginosa* deficient in *pvdA* with *S. aureus* and observed a significant but modest restoration of *S. aureus* viability compared to the WT (Fig. 5a), as previously reported (10). We then asked if the observed decrease in *pvdA* expression when alginate is overproduced, results in decreased pyoverdine production. Pyoverdine is fluorescent when excited at 400 nm, and we utilized this property to determine the amount of pyoverdine produced as measured in relative fluorescent units (RFU) per OD_600_ of *P. aeruginosa* cultures grown for 8 h under the conditions utilized in the coculture assays. The *mucA22* mutant produced approximately 40% less pyoverdine than nonmucoid, WT *P. aeruginosa*. When *algD* is deleted in the *mucA22* mutant, pyoverdine production is restored (Fig. 5b), demonstrating that pyoverdine levels are lower in alginate-producing *P. aeruginosa* strains.

### Alginate overproduction reduces HQNO production.

In addition to siderophores, we and others previously reported that 2-heptyl-4-hydroxyquinoline-*N*-oxide (HQNO) inhibits *S. aureus* respiration (7, 10, 42), and we further showed that HQNO causes a shift to a fermentative lifestyle and eventual *S. aureus* cell death during late stage biofilm growth on CFBE cells (10). In our NanoString studies described above, we did not observe a significant change in the expression of genes involved in the *Pseudomonas* quinolone signal pathway (*pqs*) in alginate-overproducing strains (open circles in Fig. 4a and b and heat map in Fig. S3a). The PqsA to -E, -L, and -H enzymes catalyze the synthesis of a series of 4-hydroxy-2-alkylquinolones (HAQs [schematic in Fig. 5c]) (15, 43). Anthranilic acid is converted by PqsA and -D to a series of precursors whose precise structures are unknown (indicated by brackets), and these intermediates are ultimately converted to either 4-hydroxy-2-heptylquinoline (HHQ) by PqsB and -C (which can be converted to 3,4-dihydroxy-2-heptylquinoline [PQS] by PqsH) or converted to HQNO by PqsL (15). Because deletion of *pqsL* reduces the ability of *P. aeruginosa* to kill *S. aureus* compared to the wild type (10) (see Fig. S4 in the supplemental material), we would have predicted that alginate overproduction might decrease expression of genes involved in HQNO synthesis, reducing the amount of HQNO produced by mucoid cells. While *P. aeruginosa* HQNO *production* peaks in late stationary phase, the *expression* of the genes encoding enzymes required for HQNO generation peaks in late exponential phase (15). For the NanoString analysis, RNA was extracted from *P. aeruginosa* cells in late stationary phase. We therefore examined the expression of *pqsL* by qRT-PCR at various time points in WT PAO1 compared to a *P. aeruginosa* PAO1 *pqsL64* mutant, previously characterized to be deficient in the production of HQNO (44). *pqsL* expression peaked for WT in late exponential phase (3 h, OD_600_ of ~1.8) (Fig. S3c), as previously observed (15). Expression of *pqsL* was then examined in the *mucA22* mutant at ~3 h and was found to be modestly but significantly decreased in relative expression compared to *mucA22 algD*::FRT (Fig. S3d). To determine if HQNO generation is altered, we measure HQNO present in culture supernatants of *P. aeruginosa* by extracting HQNO from equal cell numbers of *P. aeruginosa* strains, including the WT and the Δ*pqsA*, *mucA22*, and *mucA22 algD*::FRT mutants, and quantified HQNO by liquid chromatography coupled to mass spectrometry (LC-MS), as previously described (45). Nonmucoid WT *P. aeruginosa* produced approximately 0.80 μg/ml of HQNO, whereas the mucoid *mucA22* mutant produced 0.12 μg/ml HQNO, a significant reduction (Fig. 5d). No HQNO was detected in the Δ*pqsA* mutant, the negative-control strain. Deletion of *algD* in the *mucA22* mutant restored the production of HQNO to levels that are significantly increased compared to those of the wild type (0.98 μg/ml).

10.1128/mBio.00186-17.4 FIG S4  Alginate overproduction inhibits HQNO antimicrobial activity toward *S. aureus*. (a) *In vitro P. aeruginosa-S. aureus* coculture assays in planktonic culture with *S. aureus* JE2 only (a), *S. aureus* plus *P. aeruginosa* PAO1 (WT), (b) *S. aureus* plus *P. aeruginosa pqsL64* (c), and *P. aeruginosa mucA22* (d) in the presence of either DMSO (vehicle control [open circles]) or 12.5 µg/ml HQNO (solid circles). Log_10_ CFU/ml for *S. aureus* JE2 are indicated. Error bars indicate standard deviations from three biological replicates performed in triplicate. Download FIG S4, TIF file, 0.3 MB. Copyright © 2017 Limoli et al. 2017 Limoli et al. This content is distributed under the terms of the Creative Commons Attribution 4.0 International license.

To confirm that reduced HQNO in the *mucA22* mutant contributes to decreased killing of *S. aureus*, we asked if the addition of exogenous HQNO could restore killing of *S. aureus*. As a control, we first tested if the addition of HQNO could complement a *pqsL* mutant. As expected, *pqsL64* was unable to reduce *S. aureus* viability (Fig. 5e; Fig. S4C), supporting previous observations that HQNO is required to kill *S. aureus* (7, 10, 42). We then grew *S. aureus* in monoculture or in coculture with the *P. aeruginosa* WT, *pqsL64*, or *mucA22* strain with either 12.5 µg/ml HQNO or dimethyl sulfoxide (DMSO) (vehicle control). The addition of HQNO did not affect the viability of *S. aureus* alone or in the presence of wild-type *P. aeruginosa* PAO1 (Fig. S4a and b) but did restore killing mediated by the *pqsL64* mutant (Fig. S4c), albeit the amount of time to initiate killing was delayed compared to that with WT *P. aeruginosa*. Importantly, HQNO partially restored the ability of the *P. aeruginosa mucA22* mutant to kill *S. aureus* (Fig. S4d), suggesting decreased production of HQNO contributes to the reduced antimicrobial activity of the mucoid isolates. The addition of HQNO did not fully restore killing of *S. aureus* by the PAO1 *mucA22* strain, and the concentrations required to restore killing were higher for *mucA22* compared to *pqsL64* (Fig. 5e).

### Rhamnolipids contribute to *P. aeruginosa* killing of *S. aureus*.

**FIG 6:**
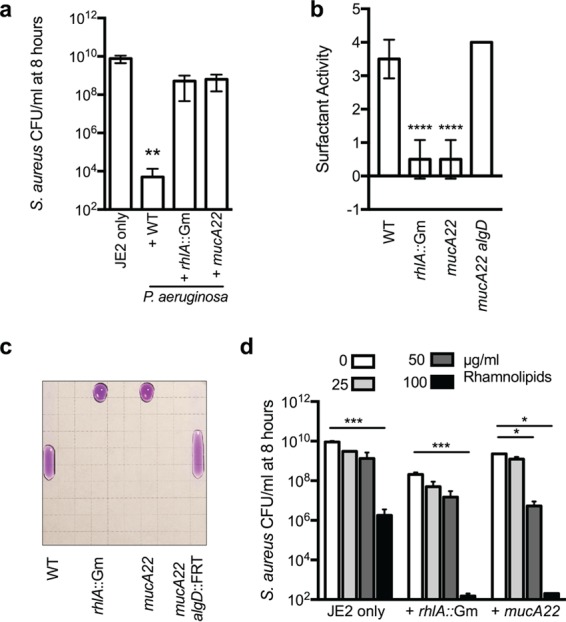
Alginate inhibits the production of rhamnolipids required to kill *S. aureus*. *In vitro P. aeruginosa-S. aureus* coculture assays in planktonic culture. In panels a and d, the log_10_ CFU/ml for *S. aureus* JE2 are indicated after 8 h of incubation, and in panels b and c, the drop collapse assay was used to measure surfactant activity. Clarified supernatants were serially diluted (1:1) with water plus 0.005% crystal violet for visualization. Twenty microliters of each dilution was spotted onto the underside of the lid of a petri plate and tilted at a 90° angle. As surfactant quantities are reduced by dilution, surface tension increases, resulting in the beading of the droplet. Surfactant scores are equal to the reciprocal of the greatest dilution at which there was surfactant activity (a collapsed drop that migrates down the plate). Quantification is indicated in panel b, and a representative image of all strains at the 1/8 dilution is shown in panel c. In panel d, competitions were performed in the presence of the indicated concentrations of rhamnolipids. Error bars indicate the standard deviation of three biological replicates performed in triplicate (two biological replicates for panel d). In panels a and b, statistical significance was determined by performing an ANOVA followed by a Dunnett’s multiple comparison test comparing each condition to JE2 in panel a and to the WT only in panel b. In panel d, statistical significance was determined by performing an ANOVA followed by a Tukey’s multiple comparison test to compare the mean survival of *S. aureus* in the presence of each rhamnolipid concentration within strains. *, *P* ≤ 0.05; **, *P* < 0.01; ***, *P* < 0.0001.

NanoString analysis of alginate-overproducing strains also revealed lower transcript levels of *rhlA* compared to those in nonmucoid strains: *rhlA* is the gene that encodes the first enzyme required for the synthesis of *P. aeruginosa* surfactants (rhamnolipids) (Fig. 4a and b). RhlA is involved in the synthesis of the fatty acid dimer 3-(3-hydroxyalkanoyloxy) alkanoic acid (HAA) moiety of rhamnolipids from 3-hydroxy fatty acid precursors, which are subsequently converted to monorhamnolipids, and dirhamnolipids, by RhlB and RhlC, respectively (46). HAA, mono-, and dirhamnolipids each have biosurfactant activity, but the type of rhamnolipid produced and the amount depends on the strain and the carbon source (47). Rhamnolipids derived from *P. aeruginosa* strains isolated from oil-contaminated soil have been shown to have antimicrobial activity against *S. aureus* (13, 14); therefore, we hypothesized that the production of rhamnolipids may also play a role in *P. aeruginosa* interactions with *S. aureus* in our model. We first confirmed reduced expression of *rhlA* in the *mucA22* mutant compared to the WT and *mucA22 algD*::FRT mutant by qRT-PCR (Fig. S3e). Furthermore, when the *rhlA* gene was disrupted in *P. aeruginosa* (PAO1 *rhlA*::Gm), the viability of *S. aureus* in coculture was similar to that seen with *mucA22* mutant (Fig. 6a).

To determine if rhamnolipid production is also decreased when alginate is overproduced, we assayed for biosurfactant activity in clarified *P. aeruginosa* supernatant from the mucoid *mucA22* mutant compared to the nonmucoid, WT, and *mucA22 algD*::FRT strains, utilizing a drop collapse assay, as previously described (48). In brief, the presence of sufficient surfactant disrupts the surface tension of droplets of bacterial supernatant on plastic, which will migrate downward when placed at a 90° angle. Supernatant from each strain (or purified rhamnolipid) was serially diluted 2-fold, and the surfactant activity calculated as 1/dilution at which the drop begins to migrate (see representative images in Fig. S5 and quantification of biological replicates in Fig. 6b). *P. aeruginosa* WT supernatant, which produces rhamnolipids, exhibits surfactant activity of approximately 3.5, compared to 0.5 when *rhlA* is disrupted (Fig. 6b). The surfactant activity of PAO1 *mucA22* phenocopies the *rhlA* mutant, and surfactant activity is restored in the *mucA22 algD* mutant, demonstrating that alginate overproduction reduces surfactant production in *P. aeruginosa*. A representative image of each strain at the 1/8 dilution in shown in Fig. 6c. We then evaluated the ability of rhamnolipids to kill *S. aureus*. We found that 100 µg/ml of a 50/50 mixture of mono- and dirhamnolipids reduces the viability of *S. aureus* from approximately 1 × 10^10^ CFU/ml to 1 × 10^6^ CFU/ml (Fig. 6d). Whereas the addition of exogenous rhamnolipids to *S. aureus* in the presence of a *rhlA* mutant or the *mucA22* mutant further reduces *S. aureus* viability to below 100 CFU/ml, which supports our findings that multiple *P. aeruginosa* antimicrobials are required to kill *S. aureus*.

10.1128/mBio.00186-17.5 FIG S5  Alginate inhibits the production of *P. aeruginosa* rhamnolipids. The results from a drop collapse assay to measure rhamnolipid-mediated surfactant activity are shown. The clarified supernatants of the indicated strains (a) and purified rhamnolipids (b [50/50 mixture of mono- and dirhamnolipids]) were serially diluted (1:1) with water plus 0.005% crystal violet (for visualization). Surfactant activity was measured by the spread of the droplet down a 90° incline. As surfactant quantities are reduced by dilution, surface tension increases, resulting in the beading of the droplet. Representative images from three biological replicates are shown. Download FIG S5, TIF file, 45.6 MB. Copyright © 2017 Limoli et al. 2017 Limoli et al. This content is distributed under the terms of the Creative Commons Attribution 4.0 International license.

## DISCUSSION

**FIG 7:**
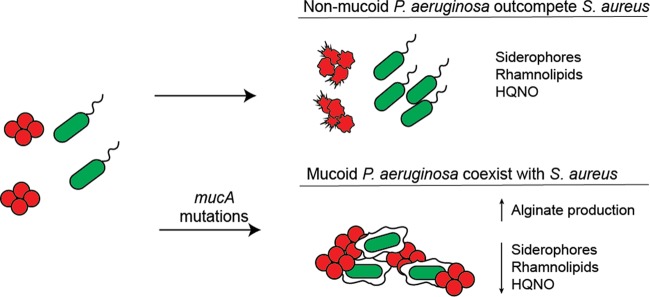
Proposed model of *P. aeruginosa* coinfection with *S. aureus*. Nonmucoid isolates produce a range of antimicrobial agents that can kill *S. aureus*, including siderophores, rhamnolipids, and HQNO, which allows *P. aeruginosa* to outcompete *S. aureus*. If *P. aeruginosa* acquires *mucA* mutations during infection leading to overproduction of the polysaccharide alginate, the expression of genes required for siderophore, HQNO, and rhamnolipid synthesis are decreased. These modifications reduce the capacity of *P. aeruginosa* to outcompete *S. aureus*, and the two species coexist in the CF lung.

From our studies, we are able to begin to generate a model of how *P. aeruginosa* and *S. aureus* may be able to coinfect the same niche in the respiratory tract of CF patients (Fig. 7). During infection *P. aeruginosa* acquires mutations in the *mucA* gene, leading to alginate overproduction. Alginate overproduction reduces the generation of siderophores, HQNO, and rhamnolipids by lowering the expression of genes encoding enzymes required for their generation. All three of these factors—HQNO, siderophores, and rhamnolipids—have documented effects in reducing the viability of *S. aureus* (7–16). These data demonstrate that the production of this polysaccharide is sufficient to block a range of antimicrobials required for reduction of *S. aureus* viability. The observations that PAO1algIND has reduced ability to kill *S. aureus* when grown in the presence of arabinose, while only producing a modest level of alginate, suggests that small changes in alginate production during infection could influence polymicrobial dynamics. In agreement with this idea, we observed increased production of both pyoverdine and HQNO compared to the WT when alginate production was completely removed by deletion of *algD* (Fig. 5b and d). Evidence that even nonmucoid *P. aeruginosa* strains produce alginate during CF pulmonary infections (41, 49) suggests that the observations in this study may be relevant beyond *P. aeruginosa* isolates that visibly produce alginate.

The correlations identified herein through studying *P. aeruginosa* isolates from coinfected CF patients support the hypothesis that one way *P. aeruginosa* and *S. aureus* coexist in the CF lung is through *P. aeruginosa* reducing antimicrobial generation, driven by alginate overproduction. While we are beginning to advance our knowledge of the distribution of microbial species during CF pulmonary infections, and how and to what extent interspecies interactions occur (50, 51), our understanding remains quite limited. We predict that in certain areas of the lung, *P. aeruginosa* and *S. aureus* are spatially segregated, which also likely contributes to *S. aureus* survival of during coinfection, independent of *P. aeruginosa* competitive phenotypes. Thus, it is necessary to further investigate the extent of spatial interspecies coexistence in CF, and its influence on microbial physiology and patient outcome. If the former hypothesis is supported, that *P. aeruginosa* adapts to the presence of competing species, we can explore if such adaptations are specific to *S. aureus*. Reported studies from the Whiteley group suggest that *P. aeruginosa* senses and responds to *N*-acetylglucosamine (GlcNAc) shed from the cell wall of Gram-positive organisms (52); therefore, some specificity of response may be afforded to Gram-positive organisms.

Our expression analysis revealed alginate-overproducing strains decrease expression of genes required for pyoverdine, HQNO, and rhamnolipid synthesis, as well as *norC*, encoding a nitric oxide reductase, and *fliC*, encoding flagellin type B. We predict alginate may also exert effects on additional *P. aeruginosa* genes not examined in this study. Each gene identified here has been reported previously to be directly or indirectly regulated by AlgT (27–30). Our data support a mechanism of indirect regulation, whereby AlgT activates transcription of P*algD*, resulting in increased alginate production, which reduces the expression of a subset of genes in the AlgT regulon. In agreement with a model of alginate-dependent regulation independent of AlgT, we did not observe changes in *algT* gene expression (or additional genes analyzed known to be AlgT regulated) in the *mucA22 algD*::FRT mutant compared to the *mucA22* strain or in PAO1algIND with and without arabinose (Fig. 3; Table S2). However, it remains a formal possibility that limiting alginate production reduces posttranscriptional AlgT activity and alters expression of only a subset of AlgT-regulated genes.

The mechanism by which alginate might reduce expression of genes required for HQNO, siderophore, and rhamnolipid biosynthesis has not been established, yet modulation of relevant *P. aeruginosa* physiology by this polysaccharide is not unprecedented. Indeed, a recent report demonstrated alginate can interfere with PQS signaling—a feature limited to alginate producers; no such inhibition of signaling was observed for the non-alginate-producing neighbors (53). Alginate has also been shown to restrict the diffusion of oxygen (54–56) and aminoglycoside antibiotics (57), and to bind and sequester reactive intermediates and iron (58). These properties may explain how alginate is able to reduce the production of multiple *P. aeruginosa* virulence factors. In the case of siderophores, alginate may help concentrate iron locally thus reducing the need to acquire iron via siderophores. Prior studies demonstrating increased alginate production during iron starvation support the hypothesis that alginate aids *P. aeruginosa* in acquiring iron (59). In this scenario, we may predict that changes in siderophore gene expression occur as part of derepression of the entire Fur regulon. However, in our NanoString studies, we did not observe a significant change in the expression of other Fur-regulated genes within the PaV2 codeset (*bfrB*, *hasR*, *phuR*, and *feoB*). Alginate might also help to sequester other secreted factors thus increasing their local concentration and feedback inhibiting their production, or making such secreted factors unavailable to the cell. Alternatively, the draw on metabolites to synthesize alginate may deplete intermediates used by other pathways. Such an explanation may inform the reduction in rhamnolipid production, as these surfactants require similar activated sugars for their synthesis. Whether via common or discrete mechanisms, it is clear that production of even a small amount of alginate results in markedly altered cellular physiology.

Our investigation of polymicrobial dynamics in CF isolates reinforces previous observations (10) that in order for *P. aeruginosa* to kill *S. aureus*, multiple secreted factors must be generated simultaneously by *P. aeruginosa*, as deletion of any one of these factors reduces *P. aeruginosa* ability to kill *S. aureus*. These antimicrobials may function individually and/or work together to enhance killing. For example, rhamnolipids have been shown to not only have antimicrobial activity, but these surfactants can increase the solubility and activity of other *P. aeruginosa* metabolites such as PQS (60). A recent report examined *S. aureus*-*P. aeruginosa* interactions in a biofilm coculture model in a flow chamber system where the *P. aeruginosa* exoproducts required for killing would be removed (61). Here the authors observed that nonmucoid *P. aeruginosa* facilitates *S. aureus* biofilm formation, whereas mucoid *P. aeruginosa* tend to outcompete *S. aureus*. This finding highlights the complexity of interspecies interactions and how environmental conditions may influence such interactions. In CF respiratory infections, microbial communities often persist in thickened, static airway secretions and in close proximity to airway epithelial cells, and the *P. aeruginosa* antimicrobials described here have not only been detected in the airway of CF patients but may be predictors of airway infection (62, 63). We therefore predict these secreted *P. aeruginosa* factors are important mediators of infection in CF respiratory disease. Finally, whether the correlation observed between reduced competition by *P. aeruginosa* and coinfection results from *P. aeruginosa* adapting in response to the presence of competing species or that reduced competition by *P. aeruginosa* occurs independently, which then permits growth of other organisms, is currently unknown.

Generation of a complete understanding of the dynamics of respiratory infections is complicated by shifting host pathogens, as well as inter- and intraspecies interactions. Here we revealed that a prevalent *P. aeruginosa* adaptation during chronic infections, mucoid conversion, correlates with reduced *P. aeruginosa* competition with *S. aureus*. Interrogation of this phenotype *in vitro* informed how mucoid conversion limits *P. aeruginosa* competition with *S. aureus*—by reducing production antistaphylococcal factors. Combining these findings with a model of coinfection during biofilm formation on CF bronchial epithelia cells allows us to gain a more complete understanding of how bacteria interact during infection.

## MATERIALS AND METHODS

### Bacterial strains and growth conditions.

The bacterial strains used in this study are listed in Table S3 in the supplemental material. *P. aeruginosa* and *Escherichia coli* were routinely grown in lysogeny broth (LB) and *S. aureus* in tryptic soy broth (TSB), with 1.5% agar for solid medium. Synthetic CF sputum medium was made as previously described (35) with 0.5% mucin. Gentamicin at 30 μg/ml and carbenicillin at 250 µg/ml were used for *P. aeruginosa*, and 100 µg/ml ampicillin was used for *E. coli* where indicated. Detailed descriptions of the construction of *P. aeruginosa* mutants can be found in Text S1 in the supplemental material.

10.1128/mBio.00186-17.9 TABLE S3  Bacterial strains and plasmids used in this study. Download TABLE S3, DOCX file, 0.1 MB. Copyright © 2017 Limoli et al. 2017 Limoli et al. This content is distributed under the terms of the Creative Commons Attribution 4.0 International license.

10.1128/mBio.00186-17.10 TABLE S4  Primers used in this study. Download TABLE S4, DOCX file, 0.1 MB. Copyright © 2017 Limoli et al. 2017 Limoli et al. This content is distributed under the terms of the Creative Commons Attribution 4.0 International license.

10.1128/mBio.00186-17.6 TEXT S1  Supplemental materials and methods. Download TEXT S1, DOCX file, 0.2 MB. Copyright © 2017 Limoli et al. 2017 Limoli et al. This content is distributed under the terms of the Creative Commons Attribution 4.0 International license.

### Coculture assays.

*P. aeruginosa* and *S. aureus* were cocultured on either agar plates, in shaking broth culture in rich medium (TSB) or synthetic CF sputum medium, or in biofilm growth on CF bronchial epithelial cells. At the indicated time points, aliquots of culture were removed, and *P. aeruginosa* and *S. aureus* were enumerated by plating on selective media (*Pseudomonas* isolation agar [PIA] and mannitol salts agar [MSA], respectively). Detailed methods are provided in Text S1.

### Alginate quantification.

Alginate was collected from cultures grown as described above for planktonic coculture assays and isolated as previously described (64), with modifications (65). Detailed protocols can be found in Text S1.

### NanoString analysis of *P. aeruginosa* transcripts.

RNA transcript levels were measured using the NanoString nCounter system (NanoString Technologies, Seattle, WA) and methods described by Geiss et al. (66). We employed a custom-designed codeset containing 75 *P. aeruginosa* genes (Table S1). The nucleotide sequences were provided to NanoString Technologies, Inc., for codeset design and construction. Each reaction mixture contained 70 ng of RNA, hybridization buffer, reporter probes, capture probes, as well as six positive and eight negative controls. RNA was hybridized with reporter and capture probes overnight at 65°C, and sample preparation ensued at the NanoString preparation station. Finally, targets were counted on the nCounter using 255 fields of view per sample. Data were analyzed using nSolver Analysis software, version 1.1 (NanoString Technologies, Seattle, WA). Raw counts were calibrated to the arithmetic mean of six spiked positive-control transcripts, and count values greater than 2 standard deviations above the average of the eight negative-control probes were considered above background. Detailed protocols for RNA isolation and quantitative real-time PCR can be found in Text S1.

### *P. aeruginosa* antimicrobial quantification.

Pyoverdine, HQNO, and rhamnolipids were quantified as previously described (45, 48, 67), respectively. Detailed protocols can be found in Text S1.

### Availability of data.

The authors certify that they will comply with mBio’s data policy: data will be made publicly available upon publication and upon request for peer review.
