# Supplementary figures and images for: Pseudomonas aeruginosa Alginate Overproduction Promotes Coexistence with Staphylococcus aureus in a Model of Cystic Fibrosis Respiratory Infection
Source: mBio. 2017 Mar 21;8(2):e00186-17. doi: 10.1128/mBio.00186-17 (PMC5362032; doi:10.1128/mBio.00186-17)

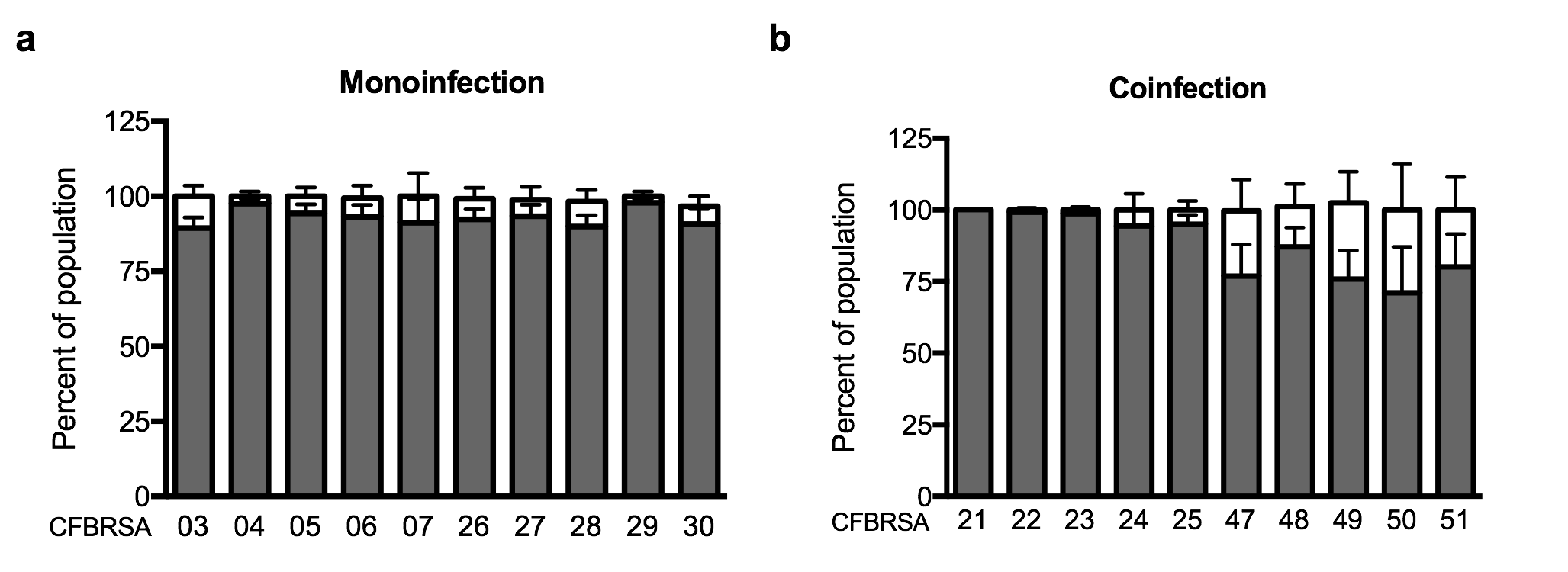

Supplement: FIG S1 [file mbo002173236sf1.tif]

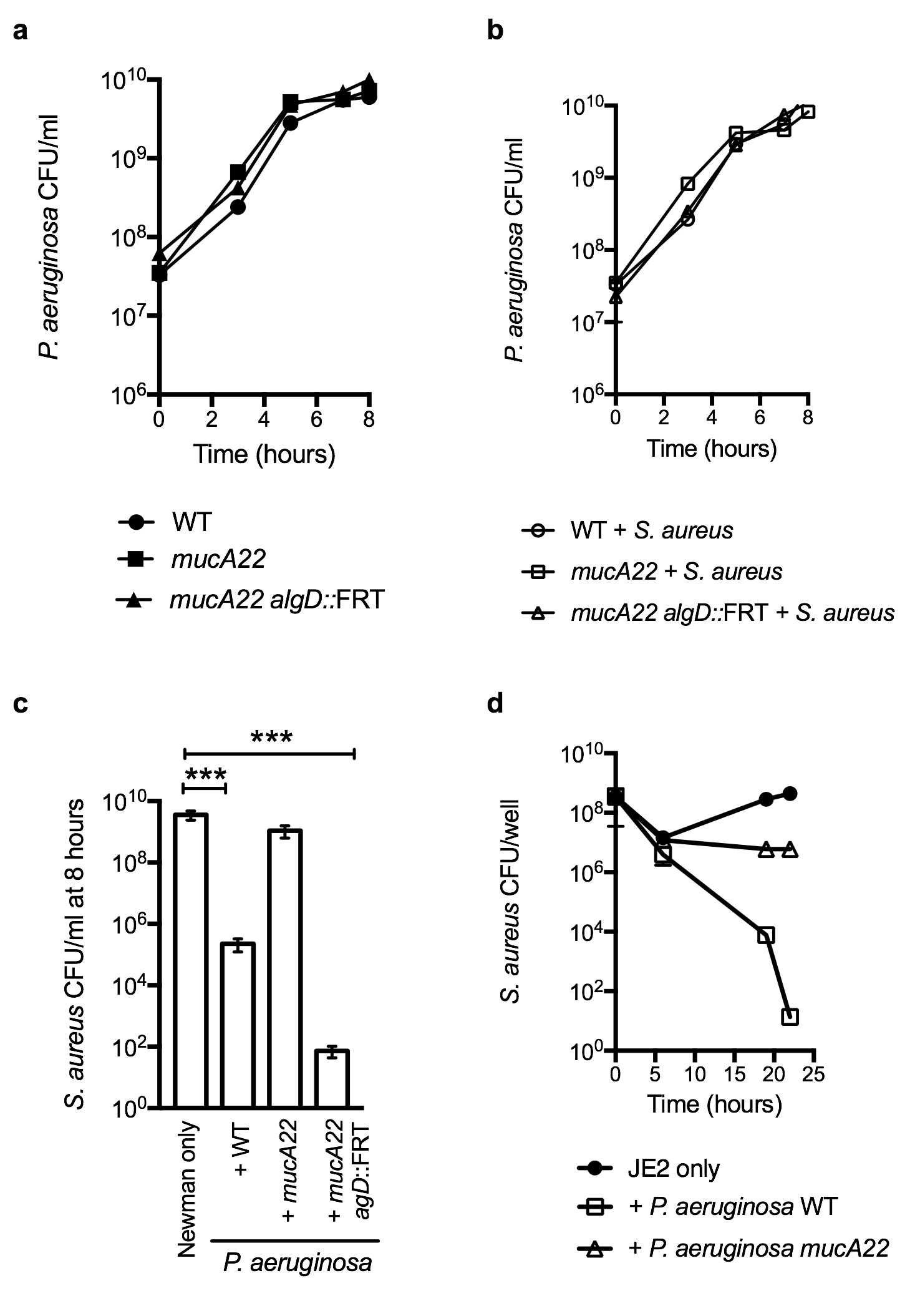

Supplement: FIG S2 [file mbo002173236sf2.tif]

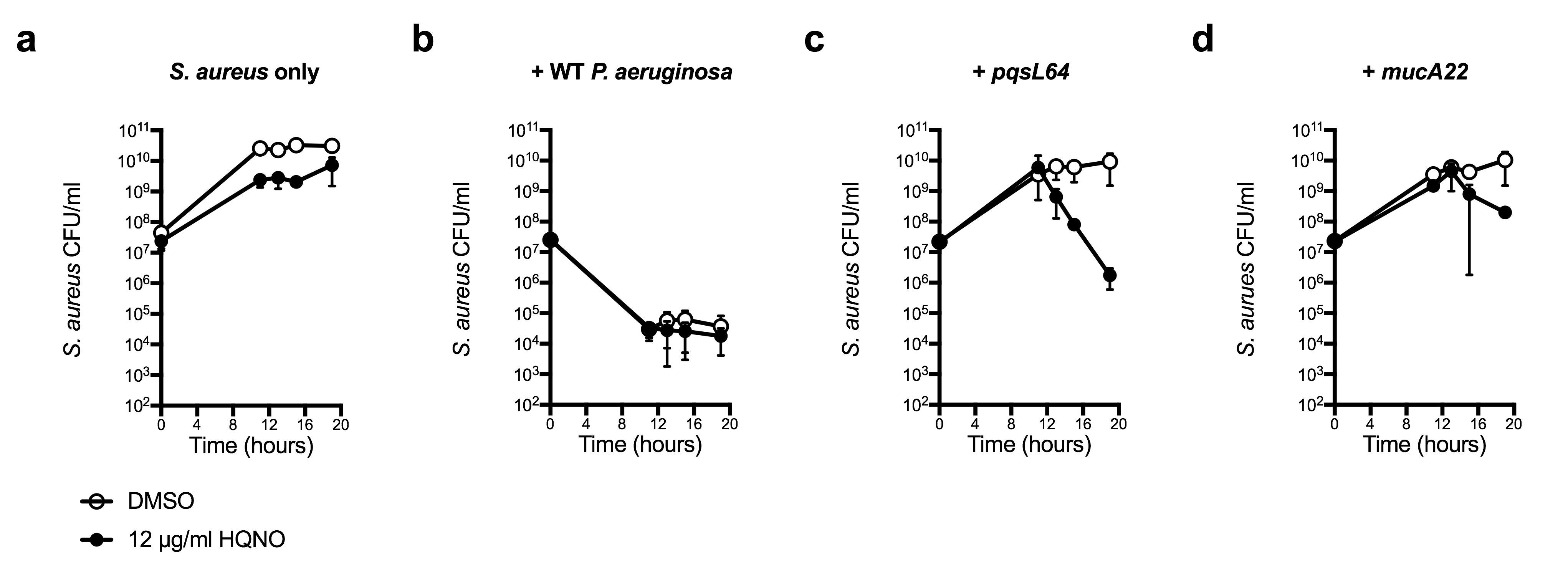

Supplement: FIG S4 [file mbo002173236sf4.tif]

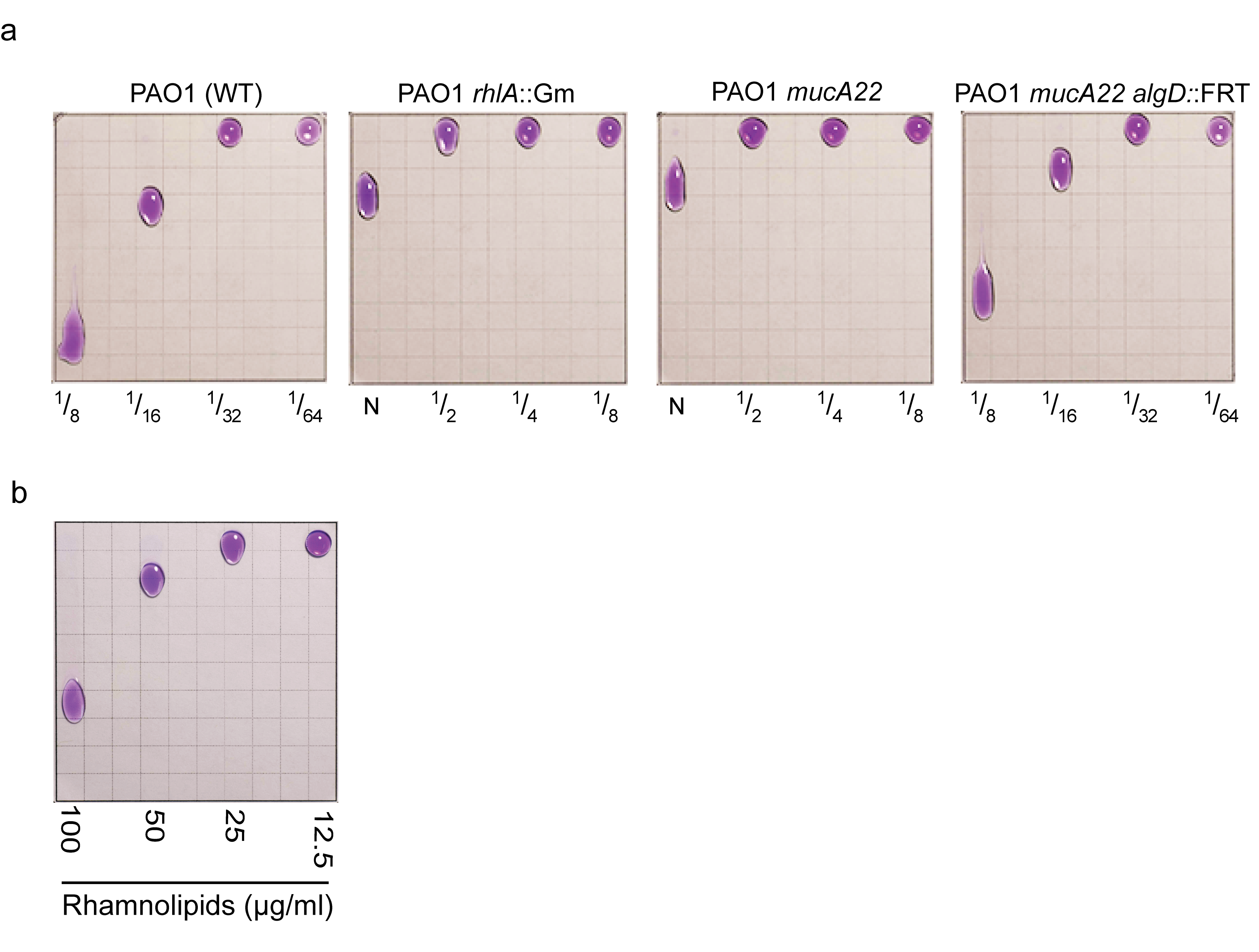

Supplement: FIG S5 [file mbo002173236sf5.tif]
