# Supplementary material for: Pseudomonas aeruginosa Alginate Overproduction Promotes Coexistence with Staphylococcus aureus in a Model of Cystic Fibrosis Respiratory Infection
Source: mBio. 2017 Mar 21;8(2):e00186-17. doi: 10.1128/mBio.00186-17 (PMC5362032; doi:10.1128/mBio.00186-17)
Supplement: TABLE S2 [file mbo002173236st2.docx]

| **Table S2. Relative expression of AlgT-regulated genes in PAV2 between PAO1 *mucA22* and PAO1 *mucA22 algD*::FRT.** | | |
| --- | --- | --- |
| Gene Name | *mucA22/mucA22 algD*::FRT^#^ | *p* value^*^ |
| *algD* | 15.14 +/- 0.84 | 0.015 |
| *algI* | 4.3 +/- 1.50 | 0.002 |
| *algT/U* | 0.90 +/- 0.90 | 0.221 |
| *flgD* | -1.67 +/- 0.63 | 0.127 |
| *flgG* | -1.58 +/- 0.92 | 0.192 |
| *flgK* | -1.41 +/- 0.66 | 0.207 |
| *hcnA* | *­-*0.85 +/- 1.16 | 0.423 |
| *plcR* | 0.50 +/- 0.98 | 0.539 |
| # log_2_ relative transcript levels for PAV2 genes were compared between PAO1 *mucA22* and PAO1 *mucA22 algD*::FRT. The mean +/- standard deviation are indicated.  **p* values were determined using an unpaired t-test followed by a two-stage linear step-up procedure of Benjamini, Krieger and Yekutieli, with Q = 1% for false discovery. | | |
