# Supplementary material for: Pseudomonas aeruginosa Alginate Overproduction Promotes Coexistence with Staphylococcus aureus in a Model of Cystic Fibrosis Respiratory Infection
Source: mBio. 2017 Mar 21;8(2):e00186-17. doi: 10.1128/mBio.00186-17 (PMC5362032; doi:10.1128/mBio.00186-17)
Supplement: TABLE S3 [file mbo002173236st3.docx]

**Table S3. Bacterial strains and plasmids used in this study.**

| **Strain or plasmid** | **Genotype or description** | **Reference or source** |
| --- | --- | --- |
| *Escherichia coli* |  |  |
| DH5α | supE44 ΔlacU169(φ80dlacZΔM15) hsdR17 thi-1 relA1 recA1 | Life Technologies |
| SM10λpir | KmR, thi-1, thr, leu, tonA, lacY, supE, recA::RP4-2-Tc::Mu, pir | Life Technologies |
|  |  |  |
| *P. aeruginosa* |  |  |
| PAO1 | Non-mucoid prototroph | (12) |
| PDO300 | PAO1 *mucA22* | (13) |
| MRD635 | PAO1 *mucA22 algD*::FRT | This study |
| MRD687 | PAO1 *algD*::FRT | This study |
| FRD1 | Prototrophic CF isolate, *mucA22* | (14) |
| FRD440 | *mucA22* His-1 algT33::TnS1 | (15) |
| FRD875 | *mucA22* *algD*::*xylE* *aacC1* | (16) |
| PA14 | Wild type, clinical isolate | (17) |
| PAO1algIND | PAO1 *ΔwspF* P*algD*::*araC*-P*ara*_BAD_ | This study |
| CFBRPA40 | Nonmucoid CF isolate | This study |
| CFBRPA43 | Mucoid CF isolate, *mucA22* | This study |
| PADHL92 | S1, Non-mucoid revertant of CFBRPA43  *mucA22*, *algT* 8 bp insertion at 138 | This study |
| PADHL93 | S2, Non-mucoid revertant of CFBRPA43  *mucA22*, C245A | This study |
| PAO1Δ*pvdA* |  | (18) |
| PJF-QA1 | PAO1 Δ*pqsA* | (19) |
| PAO1*pqsl64* | PAO1 *pqsL*64::IS*phoA*/hah-Tc | (20) |
| PAO1*rhlA*::Gm |  | (21) |
|  |  |  |
| *S. aureus* |  |  |
| JE2 | USA300 CA-Methicillin resistant strain LAC without plasmids | (22) |
| Newman | Methicillin sensitive | (23) |
|  |  |  |
| Plasmids |  |  |
| pEXAp-GW | Gateway compatible suicide vector | (5) |
| pPS856 | Gm^r^ cassette flanked by FRT | (5) |
| pENTR-D-TOPO |  | ThermoFisher |
| pFLP2 | Site-specific excision vector | (5) |
| pEX18-Gm | Gm^r^; gram negative allelic replacement vector | (4) |
