# Supplementary material for: Pseudomonas aeruginosa Alginate Overproduction Promotes Coexistence with Staphylococcus aureus in a Model of Cystic Fibrosis Respiratory Infection
Source: mBio. 2017 Mar 21;8(2):e00186-17. doi: 10.1128/mBio.00186-17 (PMC5362032; doi:10.1128/mBio.00186-17)
Supplement: TABLE S4 [file mbo002173236st4.docx]

**Tables S4. Primers used in this study.**

| **Primer Name** | **Sequence (5’-3’)** | **Source** |
| --- | --- | --- |
| oDHL05_mucAupF | TGTTGCGGGATGAGATCGAGG | (24) |
| oDHL06_mucAdnR | GGGTGGAGAAGCTGCCATTG | (24) |
| oDHL07_mucA1F21 | GGATCTTCCGCGCTCGTGAAG | (24) |
| oDHL01_algT F1 | GCATGCTTGGAGGGGAGAAC | This study |
| oDHL01_algT R1 | GTGTCAGGCTTCTCGCAACA | This study |
| GW1_pBADalgupF | TTAGGTACCATGCTCTCCGGTCTCTACGG | This study |
| algD-US-F | GGCCATTGGCAGGCATTTAAC | This study |
| algD-US-R-T1 | TCAGAGCGCTTTTGAAGCTAATTCGCGCATTCACCTCGATTGTTTG | This study |
| algD-DS-F-T2 | AGGAACTTCAAGATCCCCAATTCTGAGCCGGTAAAGAACCTCTT | This study |
| algD-DS-R | CGAGCGATAGACCATGGCAGT | This study |
| Gent-F | CGAATTAGCTTCAAAAGCGCTCTGA | (5) |
| Gent-R | CGAATTGGGGATCTTGAAGTTCCT | (5) |
| GW2_pBADalgupR | GTAGCCGTCAAGTTGTCATAAACGAACGGTAGCGCCAGGAG | This study |
| GW3_pBADalgmidF | TTATGACAACTTGACGGCTAC | This study |
| GW4_pBADalgmidR | CCAAAGATGCTGATTCGCATGAATATCCTCCTCCCATGGA | This study |
| GW5_pBADalgdownF | ATGCGAATCAGCATCTTTGG | This study |
| GW6_pBADalgdownR | GGGAAGCTTGTTGGTGCCGACGCCGAAGT | This study |
| GW7_wspFupF | CGTGAATTCTGAACGTCCTGCTGGTGCCGGA | This study |
| GW8_wspFupR | CGGGGCGATCTTTTCCAGGGACGGCATGTCATTGACGATTCCGAT | This study |
| GW9_wspFdownF | TCCCTGGAAAAGATCGCCCCG | This study |
| GW10_wspFdownR | TCTAAGCTTCTTCCTTGGTCGACAGGACGAT | This study |
| oDHL54_rpoD qPCR F1 | CGATCGGTGACGACGAAGAT | This study |
| oDHL55_rpoD qPCR R1 | GTCACATCGAACTGCTTGCC | This study |
| oDHL60_ppiD qPCR F2 | TCGATCCGAAACTGCTCCAG | This study |
| oDHL61_ppiD qPCR R2 | TTCTCGACTTCCGCCTTGTC | This study |
| oDHL62_pqsL qPCR F3 | CGGTATCGCCTCCTACGTG | This study |
| oDHL63_pqsL qPCR R3 | GGAAGCTCACCACCAGTCG | This study |
| oDHL78_rhlA qPCR F2 | GAGACCGTCGGCAAATACCT | This study |
| oDHL79_rhlA qPCR R2 | GGTGGTGTATTCGTCCCAGC | This study |
| oDHL82_pvdA qPCR F1 | TCATCGACCTCAACGACAGC | This study |
| oDHL83_pvdA qPCR R1 | GTGTTGTGGTATTCGCGCAG | This study |
