# Supplementary material for: Pseudomonas aeruginosa Alginate Overproduction Promotes Coexistence with Staphylococcus aureus in a Model of Cystic Fibrosis Respiratory Infection
Source: mBio. 2017 Mar 21;8(2):e00186-17. doi: 10.1128/mBio.00186-17 (PMC5362032; doi:10.1128/mBio.00186-17)
Supplement: TEXT S1 [file mbo002173236s1.docx]

**Supplemental Materials and Methods**

**(i) Cross-streak assay.** *P. aeruginosa* clinical isolates were challenged with *S. aureus* (USA300 LAC, JE2) and *S. aureus* clinical isolates with *P. aeruginosa* (PAO1) in an agar plate-based cross-streak assay. Single colonies of *S. aureus* and *P. aeruginosa* were cross-streaked in triplicate onto TSB + 1.5% agar according the schematic in Figure 1a, and incubated for 16 hours at 37ºC. Bacterial growth post cross-streak was scraped from the plate, resuspended in 1 ml of phosphate buffered saline (PBS), 10-fold serially diluted, and plated on *Pseudomonas isolation agar* (PIA) and mannitol salts agar (MSA) to enumerate the number of colony forming units (CFU)/ml of *P. aeruginosa* and *S. aureus* recovered, respectively. Then the CFU for each individual species was divided by the total CFU recovered (*P. aeruginosa* CFU + *S. aureus* CFU) to determine the percentage of the total population recovered for each species. The competitive index was calculated by dividing the percentage of *P. aeruginosa* by the percentage of *S. aureus* CFU. A value of 1 indicates no competitive advantage for either species.

**(ii) Planktonic coculture.** *P. aeruginosa* and *S. aureus* were grown separately overnight in TSB and diluted into warm TSB to an optical density at 600 nm (OD600) of 0.1 for each species. 12.5 ml of each species was added to a 250 ml flask (12.5 ml of TSB for monoculture) to achieve target inputs of OD_600_ = 0.05 and 0.5 x 10^8^ - 1 x 10^8^ CFU/ml for each species. Cultures were incubated at 37ºC, shaking at 225 RPM for 8 to 24 hours. At the indicated time points, aliquots of culture were removed, 10-fold serially diluted in PBS, and plated onto PIA and MSA to enumerate the number of *P. aeruginosa* and *S. aureus* CFU, respectively.

**(iii) Coculture assay on CFBE monolayers.** Coculture assays were performed as previously described (1, 2). Overnight culture of *P. aeruginosa* and *S. aureus* grown in TSB were individually centrifuged (10,000 *g*, 5 min) and washed in minimal essential medium plus 2 mM L-glutamine (MEM L-Gln). Washed bacterial cells were resuspended in MEM L-Gln to an OD600) of 0.1, and 250 µl of each species (or 250 µl MEM L-Gln for monoculture) added to triplicate wells of monolayers of CF bronchial epithelial (CFBE) airway cells in a 24-well plate. CFBE monolayers were grown as previously described (3). Target inputs were 0.5 x 10^7^ to 1 x 10^7^ CFU per well for each species in a 24-well plate. CFBE coculture plates were incubated at 37°C with 5% CO2 for 1 hour, at which point any unattached bacterial cells were removed and 500 µl of MEM L-Gln 0.4% L-arginine was added to each well and incubated for an additional 5 hours. After this additional 5 hour incubation, the planktonic cells were removed and 500 µl of fresh MEM L-Gln 0.4% L-arginine was once again replaced in each well. The established coculture biofilm was incubated for an additional 16 hours, followed by enumeration of viable cell counts. For planktonic CFU enumeration, culture supernatant was 10-fold serially diluted in PBS and plated on PIA or MSA, for *P. aeruginosa* and *S. aureus* selective growth, respectively. After removal of culture supernatants, the biofilm fraction was removed by treatment with 250 µl of 0.1% Triton X-100 in PBS, with gentle shaking for 15 min. Biofilm bacteria were then scraped and vortexed in the plate for 2 min (covered with aluminum tape), then biofilm fractions were serially diluted and plated as described for the planktonic fraction.

**Mucoid reversion.** Mucoid clinical isolate CFBRPA43 from the CF Biospecimen Registry (CFBR) at Emory University + Children’s Center for CF and Airway Disease Research was grown in 3 ml of LB, at 37ºC rolling and serially passaged into fresh medium every 12 hours. At each passage, an aliquot of culture was removed, 10-fold serially diluted in PBS, and 100 µl plated onto PIA. Single colonies identified to be non-mucoid were chosen and passaged twice more by streaking for single colonies on PIA. Non-mucoid colonies appeared after three passages and two colonies were chosen for sequence analysis of the *mucA* and *algT* genes. Genomic DNA was extracted from mucoid CFBRPA43 and the non-mucoid suppressors CFBRPA43 S1 and S2, and the *mucA* gene was amplified using the primers mucAupF and mucAdnR and *algT* using algT F1 and algT F2 (see Table S4 for a complete list of primer sequences). After verification of PCR product by agarose gel electrophoresis, PCR products were sequenced via Sanger sequencing with the primers mucAupF, mucAdnR, mucA1F21, algT F1 or algT R1, as appropriate. The sequence data produced was then aligned with the sequence of *algT* in the parental to determine if and/or where mutations occurred.

**Construction of PAO1 *mucA22 algD*::FRT.** To construct the *algD* deletion in PAO1 *mucA22*, a deletion allele was created *in vitro* and inserted into the pEXAp-GW allelic exchange vector, which is a derivative of pEX18Ap (4) with the Rfb Gateway (GW) cloning site from ThermoFisher Scientific cloned into the *Sma*I site. This was accomplished by joining three PCR products, corresponding to the regions upstream of the *algD* gene of *P. aeruginosa* PAO1, the gentamicin resistance cassette flanked by flp recombinase target sites (FRT) from pPS856 (5), and the downstream regions of *algD,* via splicing by overlap extension (SOE) PCR. Oligonucleotide primers algD-US-F and algD-US-R-T1 were used to amplify the upstream region and algD-DS-F-T2 and algD-DS-R to amplify the downstream region. Primers algD-US-R-T1 and algD-DS-F-T2 contain oligonucleotides complementary to the 5’ and 3’ ends of the gentamicin resistance gene. The gentamicin resistance cassette was amplified using the Gent-F and Gent-R primers. The *algD*::Gent fragment was cloned into pENTR-D-TOPO, then swapped it into pEXAp-GW using LR clonase (ThermoFisher Scientific), all of these procedures were done according to manufacturer's instructions.  This plasmid was transferred to PAO1 *mucA22* by electroporation. Individual colonies were patched on gentamicin and carbenicillin. Recipient strains containing chromosomal gentamicin resistance cassette flanked by FRT recombination sites were electroporated with pFLP2 plasmid (4). Transformants were streaked on LB and gentamicin to screen for excision of the gentamicin resistance cassette by Flp recombination. Gentamicin-sensitive transformants were selected on LB with 15% sucrose. Precise chromosomal recombination was verified by PCR amplification using primers specific for the regions flanking the genomic target (algD-US-F and algD-DS-R), followed by Sanger sequencing of these PCR products.

**Construction of PAO1 *ΔwspF* P*algD*::*araC*-P*ara*_BAD_ strain.** To construct clean, unmarked deletions of *wspF* in the PAO1 genome, a deletion allele was created *in vitro* and inserted into the pEX18Gm allelic exchange vector (4). To assemble this deletion allele, an in-frame segment of coding sequence constituting >95% of the gene(s) was removed from the target open reading frames (ORFs). This was accomplished by joining two PCR products, corresponding to the adjacent regions upstream and downstream of *wspF* via SOE PCR. The primers used to generate this deletion allele were flanked with *Eco*RI and *Hin*dIII restriction sites for ligation into pEX18Gm (Table S4). To generate a strain in which the *algD* operon in the PAO1 genome is under inducible control, the intergenic space between the *algD* and *PA3539 genes* (hereafter referred to as the *algD* promoter) was replaced with an *araC*-P_BAD_ promoter. The promoter replacement allele was assembled using SOE-PCR to join three PCR products, consisting of an *araC*-P*ara*_BAD_ promoter element inserted between the adjacent regions upstream and downstream of the *algD* promoter. This allele was constructed such that >95% of the *algD* promoter would be replaced with an *araC*-P*ara*_BAD_ promoter, which was fused directly to the *algD* gene preceded by an engineered ribosome binding site. This promoter replacement allele was subsequently ligated into pEX18Gm, as above.

***P. aeruginosa* gene replacement.** Gene replacement was carried out using the two-step allelic exchange method of Hmelo *et al.* (6). Briefly, allelic exchange vectors were delivered into *P. aeruginosa* PAO1 from the donor strain *E. coli* SM10 via bi-parental mating. Single-crossover mutants were selected on Vogel-Bonner minimal medium (VBMM) containing gentamicin. Double-crossover mutants were isolated on no salt LB containing 15% sucrose. Precise chromosomal recombination was verified by PCR amplification using primers specific for the regions flanking the genomic target, followed by Sanger sequencing of these PCR products. Strains with multiple gene replacements were generated by repeating this process sequentially.

**Alginate quantification.**  Alginate was collected from cultures grown as described above for planktonic coculture assays and isolated as previously described (7), with modifications (8) from the following strains: PAO1, PAO1 *mucA22*, PAO1 *mucA22 algD*::FRT, and PAO1algIND grown with and without 0.5% arabinose. Briefly, samples were mixed with equal volume of 0.85% saline, and the cells were removed by centrifugation (12,000 *g* for 30 min). The culture supernatant was mixed with equal volume of 2% cetyl pyridinium chloride, and the precipitated alginate was collected by centrifugation (12,000 *g* for 10 min at room temperature). The pellet was dissolved in 1 M NaCl, precipitated again with isopropanol, and dissolved in 0.85% saline. The concentration of alginate in solution was determined by the carbazole method described by Knutson and Jeanes (7), in which a solution of alginate (50 µl) was mixed with 200 µl of borate-sulfuric acid reagent (10 mM H_3_BO_3_ in concentrated H_2_SO_4_) and 50 µl of carbazole reagent (0.1% in ethanol). The mixture was then incubated at 100°C for 10 min, and absorbance at 550 nm was determined spectrophotometrically. The alginate concentration was determined by extrapolation from a standard curve (0 to 1 g/ml) of alginic acid from seaweed.

**RNA isolation.** RNA was isolated from *P. aeruginosa* strains during mono and coculture grown as described above for planktonic culture after 8 hours of incubation. Samples were pelleted (16,000 *g*, 5 min), and bacterial cells were lysed for 30 min at 37°C in 0.25 µg/ml lysostaphin and 2.5 µg/ml lysozyme in Tris-EDTA (TE) buffer, followed by physical lysis with 5 cycles of 30 sec beating, 30 sec on ice with a 1:1 mixture of 0.1 mm and 0.5 mm glass beads. Total RNA was isolated using TRIzol and the Direct-Zol RNA miniprep kit (Zymo Research), followed by Turbo DNA-free DNase (Life Technologies) treatment, per the manufacturer’s recommendation.

**Reverse transcription and qRT-PCR.** cDNA was synthesized from total RNA for each coculture or monoculture using the Invitrogen Superscript III first-strand synthesis system according to the manufacturer’s protocol. Briefly, total RNA (~100 ng), 10 mM deoxynucleoside triphosphates (dNTPs), and 50 ng/µl random hexamers were incubated for 5 min at 65°C in a 10 µl total volume. Following this incubation, 10 µl of cDNA synthesis mix was added for a final 20 µl reaction mixture of 1x reaction buffer, 5 mM MgCl2, 0.01 M dithiothreitol (DTT), 2 U/µl RNaseOUT, and 10 U/µl Superscript III reverse transcriptase. Reaction mixtures were incubated at 25°C for 10 min, 50°C for 50 min, 85°C for 5 min, and 4°C for 5 min. Afterward, 1 µl RNase H was added to each reaction mixture and incubated at 37°C for 20 min. Quantitative real-time PCR (qRT-PCR) was performed with three technical replicates for each sample. *P. aeruginosa*-specific primers were designed for each gene of interest and are specified in Table S4. For qRT-PCR, 1 µl of cDNA was diluted in a 10 µl reaction mixture with 0.25 M forward and reverse primers and 1µ iQ Sybr green Supermix (Bio-Rad). The qRT-PCR was as follows: 95°C for 3 min and then 95°C for 30 s, annealing temperature (55ºC) for 30 s, and 72°C for 20 s, for 40 cycles. cDNA to each gene of interest was quantified based on cycle threshold compared to a standard curve of purified *P. aeruginosa* PAO1 genomic DNA and normalized from sample to sample based on *S. aureus rpoD* quantification. *P. aeruginosa ppiD* was used as a second normalization control for initial experiments and showed results consistent with those of *rpoD*; therefore, a single gene, *rpoD*, was used for later assays and is reported here.

**HQNO quantification.** *P. aeruginosa* cultures were grown under the same conditions utilized during the planktonic coculture assays and HQNO was extracted and quantified as previously described (9). In brief, bacterial culture was mixed with the same volume of methanol containing the internal standards (20 mg/l of 5,6,7,8-tetradeutero-2-heptyl-3,4-dihydroxyquinoline and 10 mg/l of 5,6,7,8-tetradeutero-4-hydroxy-2-heptylquinoline) and then centrifuged to obtain cell-free supernatant. Analysis was performed with an Agilent HP100 coupled to a Micromass Quattro II.

**Pyoverdine quantification.** Pyoverdine was measured from culture supernatants grown under the same conditions as the planktonic coculture assay as previously described (10). Supernatants were diluted in 100 mM Tris-HCl (pH 8), and measured fluorimetrically by recording the emission at 460 nm upon excitation at 400 nm in a luminescent spectrophotometer. The relative fluorescent units (RFU) were reported per OD_600_ of bacterial culture. The background level of fluorescence measured in PAO1 Δ*pvdA* was subtracted from the experimental strains.

**Drop collapse assay.** Drop collapse assays were performed as previously described (11). Briefly, supernatants grown under the same conditions as the planktonic coculture assay were clarified by centrifugation and filtration through a 0.22 µm filter. The clarified supernatants were serially diluted (1:1) with water + 0.005% crystal violet for visualization in a 96-well plate. 20 µl of each dilution was spotted onto the underside of the lid of a petri plate and tilted at a 90º angle for 10 seconds. Surfactant activity was measured by the spread of the droplet. As surfactant quantities are reduced by dilution, surface tension increases, resulting in the beading of the droplet. Surfactant scores are equal to the reciprocal of the greatest dilution at which there was surfactant activity (a collapsed drop).

**References**

1. **Anderson GG**, **Moreau-Marquis S**, **Stanton BA**, **O'Toole GA**. 2008. *In vitro* analysis of tobramycin-treated *Pseudomonas aeruginosa* biofilms on cystic fibrosis-derived airway epithelial cells. Infect Immun **76**:1423–1433.

2. **Filkins LM, Graber JA, Olson DG, Dolben EL, Lynd LR, Bhuju S, O'Toole GA.** 2015. Coculture of *Staphylococcus aureus* with *Pseudomonas aeruginosa* drives *S. aureus* towards fermentative metabolism and reduced viability in a cystic fibrosis model. Journal of Bacteriology 197:2252–2264.

3. **Moreau-Marquis S**, **Redelman CV**, **Stanton BA**, **Anderson GG**. 2010. Co-culture models of *Pseudomonas aeruginosa* biofilms grown on live human airway cells. J Vis Exp e2186–e2186.

4. **Hoang TT**, **Karkhoff-Schweizer RR**, **Kutchma AJ**, **Schweizer HP**. 1998. A broad-host-range Flp-FRT recombination system for site-specific excision of chromosomally-located DNA sequences: application for isolation of unmarked *Pseudomonas aeruginosa* mutants. Gene **212**:77–86.

5. **Choi K-H**, **Schweizer HP**. 2005. An improved method for rapid generation of unmarked *Pseudomonas aeruginosa* deletion mutants. BMC Microbiology 2005 5:1 **5**:30.

6. **Hmelo LR**, **Borlee BR**, **Almblad H**, **Love ME**, **Randall TE**, **Tseng BS**, **Lin C**, **Irie Y**, **Storek KM**, **Yang JJ**, **Siehnel RJ**, **Howell PL**, **Singh PK**, **Tolker-Nielsen T**, **Parsek MR**, **Schweizer HP**, **Harrison JJ**. 2015. Precision-engineering the *Pseudomonas aeruginosa* genome with two-step allelic exchange. Nat Protoc **10**:1820–1841.

7. **Knutson CA**, **Jeanes A**. 1968. A new modification of the carbazole analysis: application to heteropolysaccharides. Anal Biochem **24**:470–481.

8. **Cesaretti M**, **Luppi E**, **Maccari F**, **Volpi N**. 2003. A 96-well assay for uronic acid carbazole reaction. Carbohydrate Polymers **54**:59–61.

9. **Lépine F**, **Déziel E**, **Milot S**, **Rahme LG**. 2003. A stable isotope dilution assay for the quantification of the Pseudomonas quinolone signal in *Pseudomonas aeruginosa* cultures. Biochimica et Biophysica Acta (BBA) - General Subjects **1622**:36–41.

10. **Imperi F**, **Tiburzi F**, **Visca P**. 2009. Molecular basis of pyoverdine siderophore recycling in *Pseudomonas aeruginosa*. Proc Natl Acad Sci USA **106**:20440–20445.

11. **Caiazza NC**, **Shanks RMQ**, **O'Toole GA**. 2005. Rhamnolipids Modulate Swarming Motility Patterns of *Pseudomonas aeruginosa*. Journal of Bacteriology **187**:7351–7361.

12. **Holloway BW**, **Rossiter H**, **Burgess D**, **Dodge J**. 1973. Aeruginocin tolerant mutants of *Pseudomonas aeruginosa*. Genet Res **22**:239–253.

13. **Mathee K**, **Ciofu O**, **Sternberg C**, **Lindum PW**, **Campbell JIA**, **Jensen P**, **Johnsen AH**, **Givskov M**, **Ohman DE**, **Soren M**, **Hoiby N**, **Kharazmi A**. 1999. Mucoid conversion of *Pseudomonas aeruginosa* by hydrogen peroxide: a mechanism for virulence activation in the cystic fibrosis lung. - PubMed - NCBI. Microbiology **145**:1349–1357.

14. **Ohman DE**, **Chakrabarty AM**. 1981. Genetic mapping of chromosomal determinants for the production of the exopolysaccharide alginate in a *Pseudomonas aeruginosa* cystic fibrosis isolate. Infect Immun **33**:142–148.

15. **Flynn JL**, **Ohman DE**. 1988. Cloning of genes from mucoid *Pseudomonas aeruginosa* which control spontaneous conversion to the alginate production phenotype. Journal of Bacteriology **170**:1452–1460.

16. **Woolwine SC**, **Wozniak DJ**. 1999. Identification of an *Escherichia coli pepA* homolog and its involvement in suppression of the *algB* phenotype in mucoid *Pseudomonas aeruginosa*. Journal of Bacteriology **181**:107–116.

17. **Rahme LG**, **Stevens EJ**, **Wolfort SF**, **Shao J**, **Tompkins RG**, **Ausubel FM**. 1995. Common virulence factors for bacterial pathogenicity in plants and animals. Science **268**:1899–1902.

18. **Oglesby-Sherrouse AG**, **Djapgne L**, **Nguyen AT**, **Vasil AI**, **Vasil ML**. 2014. The complex interplay of iron, biofilm formation, and mucoidy affecting antimicrobial resistance of *Pseudomonas aeruginosa*. Pathogens Disease **70**:307–320.

19. **Farrow JM**, **Sund ZM**, **Ellison ML**, **Wade DS**, **Coleman JP**, **Pesci EC**. 2008. PqsE functions independently of PqsR-Pseudomonas quinolone signal and enhances the *rhl* quorum-sensing system. Journal of Bacteriology **190**:7043–7051.

20. **D'Argenio DA**, **Calfee MW**, **Rainey PB**, **Pesci EC**. 2002. Autolysis and autoaggregation in *Pseudomonas aeruginosa* colony morphology mutants. Journal of Bacteriology **184**:6481–6489.

21. **Rahim R**, **Ochsner UA**, **Olvera C**, **Graninger M**, **Messner P**, **Lam JS**, **Soberón-Chávez G**. 2001. Cloning and functional characterization of the *Pseudomonas aeruginosa* *rhlC* gene that encodes rhamnosyltransferase 2, an enzyme responsible for di-rhamnolipid biosynthesis. Mol Microbiol **40**:708–718.

22. **McClure CD**, **Schiller NL**. 1996. Inhibition of macrophage phagocytosis by *Pseudomonas aeruginosa* rhamnolipids in vitro and in vivo. Curr Microbiol **33**:109–117.

23. **Duthie ES**. 1952. Variation in the antigenic composition of staphylococcal coagulase. J Gen Microbiol **7**:320–326.

24. **Limoli DH**, **Rockel AB**, **Host KM**, **Jha A**, **Kopp BT**, **Hollis T**, **Wozniak DJ**. 2014. Cationic antimicrobial peptides promote microbial mutagenesis and pathoadaptation in chronic infections. PLoS Pathog **10**:e1004083.
